# Tetra-, penta-, and hexa-nor-lanostane triterpenes from the medicinal fungus *Ganoderma australe*

**DOI:** 10.1007/s13659-022-00356-x

**Published:** 2022-08-16

**Authors:** Lin Zhou, Subiy Akbar, Meng-Xi Wang, He-Ping Chen, Ji-Kai Liu

**Affiliations:** School of Pharmaceutical Sciences, South-Central Minzu University, 430074 Wuhan, China

**Keywords:** Basidiomycete, *Ganoderma australe*, Nor-lanostane, Structural determination, Anti-NO production

## Abstract

**Abstract:**

Chemical investigation on the medicinal fungus *Ganoderma australe* led to the identification of ten new nor-lanostane triterpenes, namely two hexa-nor ones, ganoaustratetraenones A (**1**) and B (**2**), five penta-nor ones, ganoaustraldehydes A–E (**3**–**7**), and three tetra-nor ones ganoaustrenoic acids A–C (**8**–**10**). The chemical structures along with the absolute configurations were determined by extensive spectroscopic analysis of 1D & 2D NMR and HRESIMS data. The postulated biosynthesis pathways of these compounds were proposed. Ganoaustraldehydes A (**3**) and B (**4**) showed moderate inhibition against nitric oxide production in RAW264.7 macrophage cells with the respective IC_50_ values of 32.5, 34.2 *µ*M (the IC_50_ of positive control pyrrolidine dithiocarbamate was 20.0 *µ*M).

**Graphical Abstract:**

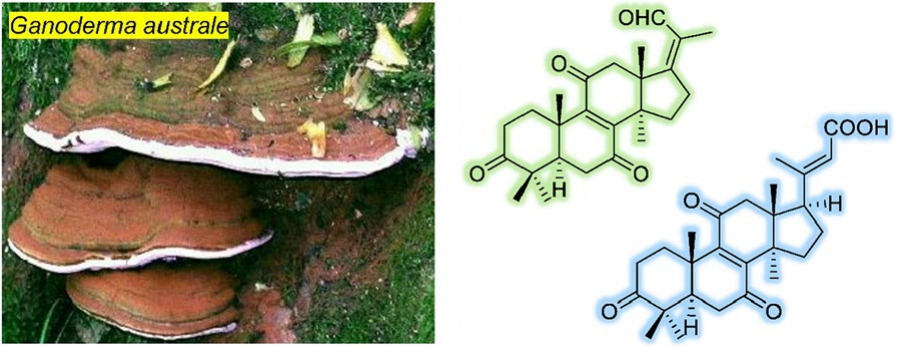

**Supplementary Information:**

The online version contains supplementary material available at 10.1007/s13659-022-00356-x.

## Introduction

The genus *Ganoderma* contains more than 80 species of wood decaying fungi mostly distributed in tropical and subtropical areas. Different species of *Ganoderma* have been used in Traditional Chinese Medicine for thousands of years for the treatment of many kinds of diseases, for example, hypertension, respiratory diseases, gastrointestinal disorders, autoimmune diseases [1]. The two species, *G. lucidum* and *G. sinense* were recorded in the recent five editions of *Chinese Pharmacopoeia* with the name “Lingzhi” from the year 2000. Numerous studies have shown that polysaccharides and the main secondary metabolites–triterpenoids, are responsible for the biological activities of *Ganoderma*, such as immunoregulatory, antiviral, hepatoprotective effects [2–7]. Besides, meroterpenes (farnesyl hydroquinones), the other main constituent in *Ganoderma*, have attracted much attention in recent years [8, 9]. *Ganoderma* triterpenes are always characterized by structural poly-oxygenations [6]. Among the reported structures, the popular positions been oxygenated are C-3, C-7, C-11, C-15, C-23, and C-26. The oxygenations in the side chain sometimes trigger the C-C bond cleavage by retro-aldol reaction or oxidative cleavage to yield nor-lanostane, a minor group of *Ganoderema* triterpenes, such as C24 (hexa-nor), C27 (tri-nor), and C-25 (penta-nor) lanostanes [5, 6].

*Ganoderma australe* is regarded as an alternative of the official-recognized species *G. lucidum*, and is used as a folk medicine in some ethnic minority areas of Yunnan Province, China. This fungus, however, was chemically under-investigated compared to other easily widely used *Ganoderma* species, such as *G. lucidum*, *G. cochlear*, and *G. sinense*. Previous studies on the secondary metabolites of *G. australe* have led to the isolation of some lanostane triterpenes [10–13], meroterpenoids [14, 15], and alkaloids [14]. In this study, we would like to report ten new nor-lanostane triterpenes isolated from *G. australe*, of which the structures are assigned by extensive NMR and HRESIMS spectroscopic analysis. The new structures are classified into tetra-nor-, penta-nor-, and hexa-nor-lanostanes, and are featured by C-6 oxygenation or *α*,*β*-unsaturated aldehyde groups, which are unusual modifications in the reported *Ganoderma* triterpenes, thereby inspiring a biosynthetic proposal of these compounds.

## Results and discussion

### Structure elucidation of compounds 1–10

**Fig. 1:**
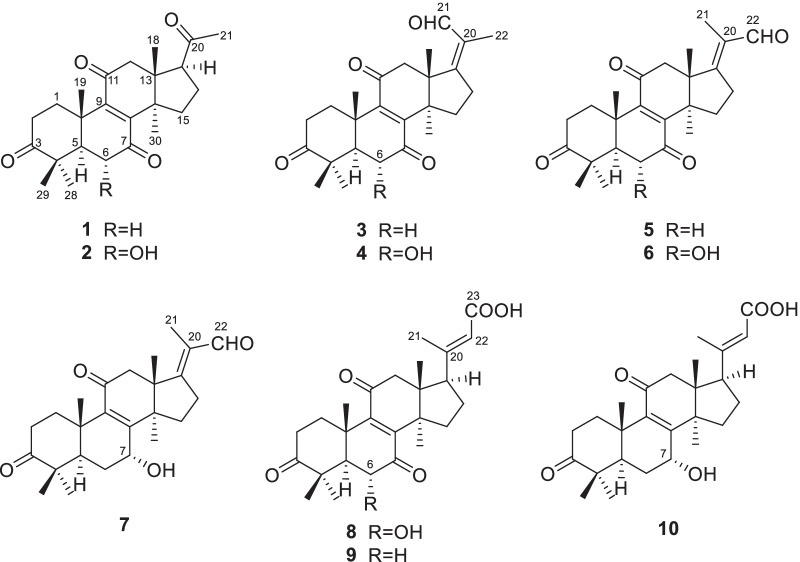
Structures of compounds **1**–**10**

**Fig. 2:**
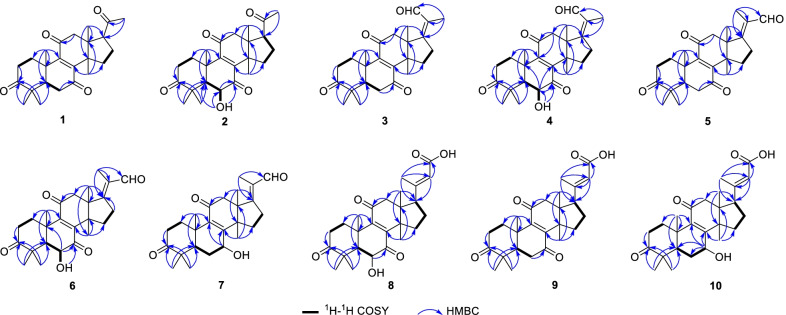
Key ^1^H-^1^H COSY and HMBC correlations of compounds **1**–**10**

Ganoaustratetraenone A (**1**) (Fig. 1), isolated as pale-yellow needles, has the molecular formula of C_24_H_32_O_4_, which was determined from the HRESIMS analysis (*m/z* 385.23724 [M + H]^+^, calcd. for C_24_H_33_O_4_, 385.23718) (Additional file: 1). The 1D NMR spectroscopic data of **1** (Tables 1 and 2) displayed six singlet methyls, six methylenes, two methines, four quaternary carbons (*sp*^3^ hybridized), a pair of non-protonated olefinic carbons, and four ketone carbonyls. The ^1^H and ^13^C NMR data of **1** showed high similarity to those of the previously described compound 4,4,14*α*-trimethyl-3,7,11,15,20-pentaoxo-5*α*-pregn-8-en [16], a semisynthetic product of methyl ganoderate O by alkaline treatment. There was only one difference between the structure of ganoaustratetraenone A and 4,4,14*α*-trimethyl-3,7,11,15,20-pentaoxo-5*α*-pregn-8-en, which was revealed by analyzing the NMR spectra. The pivotal HMBC correlation from H_3_-30 (*δ*_H_ 1.27, s) to a methylene at *δ*_C_ 32.3 (Fig. 2) suggested that C-15 of **1** is a methylene instead of being a ketone group in 4,4,14*α*-trimethyl-3,7,11,15,20-pentaoxo-5*α*-pregn-8-en. Therefore, compound **1** was elucidated to be a hexa-nor-lanostane derivative.

**Table 1:** ^1^ H NMR spectroscopic data for compounds **1**–**5**

No.	1^*a*^	2^*a*^	3^*b*^	4^*a*^	5^*a*^
1	2.95, overlapped	2.85, ddd (14.0, 9.6, 7.5)	2.89, ddd (14.5, 8.6, 6.2)	2.87, ddd (17.2, 9.9, 7.4)	2.98, overlapped
	1.73, overlapped	1.84, ddd (14.0, 13.5, 2.6)	1.89, overlapped	1.91, br. dd (17.2, 15.2)	1.77, overlapped
2	2.60, ddd (15.0, 9.3, 6.0)	2.74, ddd (14.8, 13.5, 7.5)	2.71, overlapped	2.76, overlapped	2.63, ddd (15.5, 9.5, 5.8)
	2.50, overlapped	2.34, ddd (14.8, 9.6, 2.6)	2.45, overlapped	2.35, overlapped	2.52, overlapped
5	2.24, m	2.30, d (13.6)	2.41, overlapped	2.34, overlapped	2.31, dd (14.7, 2.7)
6	2.53, t (14.4)	4.42, dd (13.6, 3.0)	2.73, overlapped	4.47, dd (13.6, 3.2)	2.58, br. t (14.7)
	2.40, dd (14.4, 2.5)		2.38, dd (12.7, 2.3)		2.44, dd (14.7, 2.6)
12	2.91, dd (16.4, 1.1)	2.93, d (16.9)	3.31, d (16.3)	3.21, d (16.8)	3.10 d (16.8)
	2.70, d (16.4)	2.73, d (16.9)	3.11, d (16.3)	3.08, d (16.8)	3.05 d (16.8)
15	2.26, overlapped	2.26, overlapped	2.46, overlapped	2.60, dd (19.5, 10.0)	2.52, overlapped
	1.75, overlapped	1.68, ddd (19.6, 12.1, 7.2)	1.93, overlapped	2.48, dd (12.8, 8.8)	1.91, td (13.2, 9.8)
16	2.26, overlapped	2.30, overlapped	2.73, overlapped	2.70, dd (19.5, 8.8)	3.12, m
	1.87, overlapped	1.90, overlapped	2.62, dd (19.3, 9.3)	1.80, br. dd (12.8, 10.0)	2.92, overlapped
17	2.98, overlapped	3.02, dd (9.2, 9.2)			
18	0.75, s	0.75, s	1.21, s	1.17, s	1.07, s
19	1.28, s	1.24, s	1.29, s	1.25, s	1.29, s
21	2.13, s	2.13, s	10.00, s	10.03, s	1.80, t (1.6)
22			1.74, s	1.76, s	10.00, s
28	1.12, s	1.34, s	1.14, s	1.36, s	1.15, s
29	1.10, s	1.43, s	1.12, s	1.45, s	1.12, s
30	1.27, s	1.35, s	1.25, s	1.30, s	1.26, s
6–OH		3.68, d (3.0)		3.69, d (3.2)	

^a^Measured at 600 MHz (CDCl_3_); ^b^Measured at 500 MHz (CD_3_OD)

**Table 2:** ^13^C NMR spectroscopic data for compounds **1**–**10**

No.	1^*a*^	2^*a*^	3^*b*^	4^*a*^	5^*a*^	6^*a*^	7^*a*^	8^*a*^	9^*a*^	10^*c*^
1	35.0, CH_2_	35.3, CH_2_	36.2, CH_2_	35.2, CH_2_	35.0, CH_2_	35.2, CH_2_	34.0, CH_2_	35.3, CH_2_	35.1, CH_2_	34.2, CH_2_
2	34.1, CH_2_	33.4, CH_2_	35.1, CH_2_	33.3, CH_2_	34.1, CH_2_	33.3, CH_2_	34.7, CH_2_	33.4, CH_2_	34.1, CH_2_	34.9, CH_2_
3	215.6, C	216.7, C	218.8, C	216.5, C	215.6, C	216.6, C	217.7, C	216.7, C	215.7, C	218.0, C
4	46.8, C	47.7, C	48.0, C	47.8, C	46.9, C	47.8, C	46.4, C	48.0, C	46.8, C	46.5, C
5	49.8, CH	54.9, CH	50.9, CH	54.9, CH	49.8, CH	54.9, CH	44.9, CH	54.9, CH	49.8, CH	45.2, CH
6	37.0, CH_2_	72.1, CH	38.0, CH_2_	72.3, CH	37.2, CH_2_	72.2, CH	29.7, CH_2_	72.2, CH	37.1, CH_2_	29.4, CH_2_
7	201.1, C	202.6, C	202.9, C	202.5, C	201.1, C	202.6, C	67.0, CH	202.6, C	201.2, C	67.3, CH
8	150.7, C	148.0, C	151.5, C	147.4, C	149.9, C	147.2, C	158.1, C	148.3, C	151.0, C	159.5, C
9	150.2, C	150.1, C	151.0, C	149.8, C	150.3, C	150.2, C	140.4, C	150.1, C	150.2, C	140.2, C
10	39.0, C	39.9, C	40.3, C	40.0, C	39.1, C	40.0, C	37.8, C	39.9, C	39.0, C	38.0, C
11	200.7, C	199.6, C	201.5, C	198.7, C	200.6, C	199.5, C	198.8, C	200.0, C	201.0, C	199.5, C
12	49.9, CH_2_	49.8, CH_2_	52.8, CH_2_	51.9, CH_2_	49.3, CH_2_	49.2, CH_2_	49.1, CH_2_	49.9, CH_2_	49.9, CH_2_	50.3, CH_2_
13^*d*^	47.3, C	47.0, C	49.0, C	47.7, C	46.5, C	46.2, C	48.6, C	47.7, C	48.3, C	48.6, C
14^*d*^	48.8, C	48.5, C	53.7, C	51.9, C	52.9, C	52.6, C	53.0, C	48.0, C	48.4, C	50.7, C
15	32.2, CH_2_	31.7, CH_2_	31.7, CH_2_	30.2, CH_2_	31.2, CH_2_	30.8, CH_2_	29.2, CH_2_	31.8, CH_2_	32.4, CH_2_	30.4, CH_2_
16	21.8, CH_2_	21.8, CH_2_	31.5, CH_2_	30.4, CH_2_	26.3, CH_2_	26.3, CH_2_	26.3, CH_2_	23.3, CH_2_	23.3, CH_2_	23.2, CH_2_
17	57.2, CH	57.2, CH	170.2, C	166.4, C	165.5, C	164.8, C	166.4, C	53.3, CH	53.3, CH	54.3, CH
18	18.6, CH_3_	18.7, CH_3_	25.1, CH_3_	25.0, CH_3_	21.5, CH_3_	21.6, CH_3_	21.4, CH_3_	18.6, CH_3_	18.5, CH_3_	18.7, CH_3_
19	18.1, CH_3_	19.5, CH_3_	18.8, CH_3_	19.8, CH_3_	18.3, CH_3_	19.8, CH_3_	17.9, CH_3_	19.5, CH_3_	18.1, CH_3_	17.7, CH_3_
20	208.7, C	208.3, C	133.0, C	132.5, C	128.9, C	129.9, C	129.2, C	161.9, C	161.6, C	162.2, C
21	31.4, CH_3_	31.3, CH_3_	192.1, CH	189.8, CH	9.6, CH_3_	9.7, CH_3_	9.6, CH_3_	21.1, CH_3_	21.2, CH_3_	21.1, CH_3_
22			12.2, CH_3_	12.3, CH_3_	193.0, CH	192.8, CH	192.8, CH	116.9, CH	116.5, CH	115.8, CH
23								171.2, C	170.3, C	170.2, C
28	27.5, CH_3_	31.2, CH_3_	27.9, CH_3_	31.2, CH_3_	27.6, CH_3_	31.2, CH_3_	27.6, CH_3_	31.2, CH_3_	27.6, C	27.8, CH_3_
29	20.5, CH_3_	19.4, CH_3_	20.8, CH_3_	19.3, CH_3_	20.4, CH_3_	19.3, CH_3_	20.5, CH_3_	19.4, CH_3_	20.5, C	20.6, CH_3_
30	26.0, CH_3_	26.1, CH_3_	27.2, CH_3_	27.2, CH_2_	27.4, CH_3_	27.4, CH_3_	29.2, CH_3_	26.4, CH_3_	26.3, C	27.8, CH_3_

^a^Measured at 150 MHz (CDCl_3_); ^b^Measured at 125 MHz (CD_3_OD); ^c^Measured at 200 MHz (CDCl_3_);^d^The data were assigned with the aid of software estimation

**Fig. 3:**
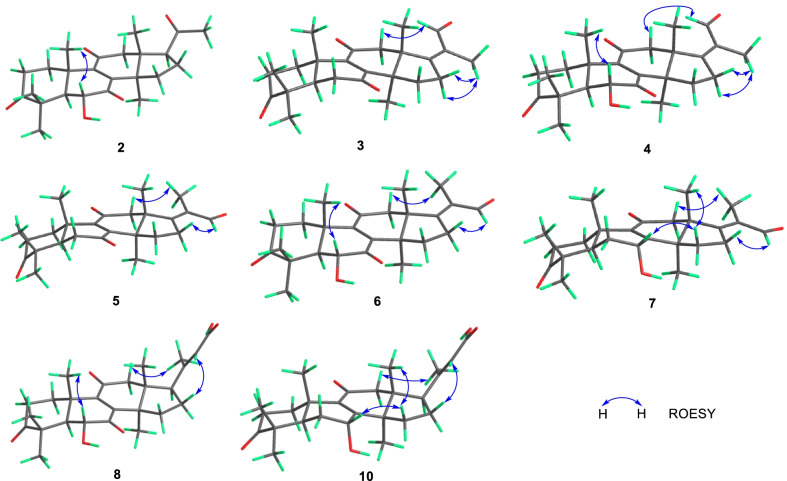
Key ROESY correlations of compounds **2**–**8**, and **10**

Compound **2** (Fig. 1) was isolated as a white powder. The HRESIMS analysis of **2** gave a protonated ion peak at *m/z* 401.23224 [M + H]^+^, corresponding to the molecular formula of C_24_H_32_O_5_ (calcd. for C_24_H_33_O_5_, 401.23280) (Additional file: 1). The 1D NMR spectroscopic data of **2** (Tables 1 and 2) showed six singlet methyls, five methylenes, three methines including one oxygenated, four quaternary carbons (*sp*^3^ hybridized), a pair of non-protonated olefinic carbons, and four ketone carbonyls. Comparing the NMR data of **1** and **2** suggested that **2** was a structural congener of **1**. Compound **2** differed from **1** by the presence of a hydroxy substituent at C-6, which was confirmed by the chemical shift of C-6 (*δ*_C_ 72.1), by the ^1^H-^1^H COSY correlation of H-5 (*δ*_H_ 2.30)/H-6 (*δ*_H_ 4.42), and by the key HMBC correlations from OH-6 (*δ*_H_ 3.68) to C-5 (*δ*_C_ 54.9), C-6, and C-7 (*δ*_C_ 202.6) (Fig. 2). The ROESY correlation of H_3_-19 (*δ*_H_ 1.24)/H-6 enabled the assignment of OH-6 as *α* orientation (Fig. 3). Thus, compound **2** was determined to be 4,4,14*α*-trimethyl-6*α*-hydroxy-5*α*-pregn-8-en-3,7,11,20-tetraone, and was trivially named ganoaustratetraenone B.

Ganoaustraldehyde A (**3**) (Fig. 1) was obtained as a white, amorphous powder. The 1D NMR spectroscopic data of **3** showed six methyl singlets, six methylenes, one methine, four quaternary carbons (*sp*^3^ hybridized), two pairs of non-protonated olefinic carbons, three ketone carbonyls, and an aldehyde carbon. The NMR data of **3** (Tables 1 and 2) showed similarity to those of ganodernoid A [17], a penta-nor-lanostane triterpenes isolated from *Ganoderma lucidum*. Analysis of the HMBC, ^1^H-^1^H COSY, and ROESY spectra of **3** allowed the elucidation of the chemical structure, and also revealed the differences between **3** and ganodernoid A. The key HMBC correlations from H_3_-30 (*δ*_H_ 1.25) to C-15 (*δ*_C_ 31.7), from H_3_-18 (*δ*_H_ 1.21) to C-17 (*δ*_C_ 170.2), and from H_3_-22 (*δ*_H_ 1.74) to C-17, C-20 (*δ*_C_ 133.0), and C-21 (*δ*_C_ 192.1) (Fig. 2) indicated that the C-15 in **3** was a methylene group, C-21 was an aldehyde group, and C-17 and C-20 was a double bond. Therefore, the 2D structure of **3** was elucidated as shown in Fig. 1. The key ROESY correlations between the aldehyde proton at *δ*_H_ 10.00 and H-12 (*δ*_H_ 3.11) (Fig. 3) suggested that the C-17-C-21 double bond was *Z* configuration. The above assignment is consistent with the molecular formula of **3**, C_25_H_32_O_4_, which was determined from the HRESIMS analysis (ion peak at *m/z* 397.23721 [M + H]^+^, calcd. for C_25_H_33_O_4_, 397.23734) (Additional file: 1).

The white amorphous powder ganoaustraldehyde B (**4**) (Fig. 1) returned a protonated ion peak at *m/z* 413.23221 [M + H]^+^ in the HRESIMS analysis, suggesting the molecular formula of C_25_H_32_O_5_ (calcd. for C_25_H_33_O_5_, 413.23225) (Additional file: 1). The 1D NMR spectroscopic data of **4** (Tables 1 and 2) highly resembled to those of compound **3**, indicating the analogous structures of **3** and **4**. The signal of an oxymethine at *δ*_C_ 72.3 (C-6) in the ^13^C NMR spectra of **4** compared to those of **3**, along with the 2D NMR correlations of **4**, including the ^1^H-^1^H COSY correlations between H-5 (*δ*_H_ 2.34) and H-6 (*δ*_H_ 4.47), and HMBC correlations from 6-OH (*δ*_H_ 3.69) to C-6 (*δ*_C_ 72.3) and C-7 (*δ*_C_ 202.5) (Fig. 2) suggested that there was a hydroxy group substituted at C-6 in **4**. The stereochemistry of the C-17-C-21 double bond was assigned as *Z* configuration according to the ROESY cross peaks between H-21 (*δ*_H_ 10.03) and H-12 (*δ*_H_ 3.08) (Fig. 3). The OH-6 was determined to be *α* configuration by the diagnostic ROESY signals of H-6/H_3_-19 (*δ*_H_ 1.25) (Fig. 3). Therefore, compound **4** was elucidated as shown in Fig. 1.

The molecular formula of compounds **5** and **6** (Fig. 1) were same with those of **3** and **4**, respectively. The 1D NMR data of **5** and **6** (Tables 1, 3 and 2) also exhibited high similarity to those of **3** and **4**, respectively, thus indicating that **5** and **6** were the respective structure congeners of **3** and **4**. Analysis of the 2D NMR spectra of **5** and **6** enabled us to identify the only difference between these two pairs of compounds, which was the configuration of C-17-C-21 double bond (Fig. 2). The diagnostic ROESY correlations of H_3_-21(*δ*_H_ 1.80)/H-12 (*δ*_H_ 3.05, 3.10), and H-22 (*δ*_H_ 10.00)/H-16 (*δ*_H_ 3.12 in **5**; 3.19 in **6**) (Fig. 3) suggested the *E* configuration of C-17-C-21 double bonds of **5** and **6** (Fig. 3). The configuration of OH-6 of **6** was determined as *α* orientation by the ROESY correlation of H-6/H_3_-19 (Fig. 3). Therefore, compounds **5** and **6** were identified as ganoaustraldehydes C and D, respectively.

**Table 3:** ^1^H NMR spectroscopic data of compounds **6**−**10** (600 MHz, CDCl_3_)

No.	6	7	8	9	10
1	2.88, m	2.62, ddd (15.7, 9.6, 5.9)	2.84, ddd (14.1, 9.6, 7.4)	2.96, ddd (14.3, 8.4, 6.2)	2.60, ddd (15.2, 9.7, 5.9)
	1.88, ddd (17.1, 10.0, 7.5)	2.47, ddd (15.7, 8.4, 6.2)	1.84, ddd (14.1, 12.1, 2.2)	1.74, ddd (14.3, 9.3, 6.8)	2.45, ddd (15.2, 8.3, 6.5)
2	2.75, ddd (17.1, 12.5, 7.5)	2.99, ddd (14.2, 8.4, 5.9)	2.75, ddd (14.8, 12.1, 7.4)	2.61, ddd (15.6, 9.3, 6.2)	2.97, ddd (14.3, 8.3, 5.9)
	2.34, overlapped	1.75, ddd (14.2, 9.6, 6.2)	2.34, ddd (14.8, 9.6, 2.2)	2.51, ddd (15.6, 8.4, 6.8)	1.70, ddd (14.3, 9.7, 6.5)
5	2.34, overlapped	2.12, overlapped	2.30, d (13.6)	2.26, dd (15.0, 2.4)	2.09, dd (11.8, 3.8)
6	4.46, dd (13.6, 3.2)	1.74, overlapped	4.43, d (13.6)	2.55, dd (15.0, 14.5)	1.70, overlapped
		1.24, overlapped		2.40, dd (14.5, 2.4)	1.25, overlapped
7		4.52, br. t (3.3)			4.48, br. t (3.0)
12	3.10, d (17.0)	3.04, d (17.2)	2.82, d (17.7)	2.80, d (16.4)	2.78, d (17.4)
	3.05, d (17.0)	2.89, d (17.2)	2.52, d (17.7)	2.51, d (16.4)	2.40, d (17.4)
15	2.48, br. dd (13.3, 9.3)	2.14, overlapped	2.27, m	2.29, overlapped	2.04, m
	1.83, overlapped	2.05, m	1.71, m	1.81, m	1.85, m
16	3.19, br. dd (18.6, 9.4)	3.18, m	1.96, overlapped, 2H	1.96, m	2.04, overlapped
	2.98, br. dd (18.6, 10.0)	3.02, m		1.90, m	1.95, m
17			2.87, overlapped	2.85, dd (9.1, 9.1)	2.92, overlapped
18	1.06, s	1.04, s	0.70, s	0.71, s	0.68, s
19	1.25, s	1.05, s	1.23, s	1.28, s	1.03, s
21	1.80, t (1.7)	1.79, s	2.17, s	2.18, s	2.17, s
22	10.00, s	10.01, s	5.77, s	5.78, s	5.78, s
28	1.36, s	1.16, s	1.34, s	1.14, s	1.14, s
29	1.45, s	1.08, s	1.44, s	1.11, s	1.07, s
30	1.32, s	1.30, s	1.36, s	1.30, s	1.36, s
6–OH	3.69, d (3.2)				

Compound **7** (Fig. 1), a pale-yellow oil, had a protonated ion peak at *m/z* 399.25305 in the HRESIMS analysis (calcd. for C_25_H_35_O_4_, 399.25353) (Additional file: 1). Analysis of the ^1^H and ^13^C NMR spectroscopic data of **3** (Tables 3 and 2) revealed that this compound was a structural analogue of **5**. The main difference between the two analogues was C-7. In the HMBC spectrum of compound **7**, significant correlations from H-7 (*δ*_H_ 4.52) to C-8 (*δ*_C_ 158.1) and C-9 (*δ*_C_ 140.4) were observed (Fig. 2). This evidence together with the ^1^H-^1^H COSY correlation of H-5/H-6/H-7 (Fig. 2) suggested that C-7 in **7** was an oxygenated methine, instead of being a ketone group in **5**. The exocyclic C-17-C-21 double bond was assigned as *E* configuration by the key ROESY correlations of H_3_-21 (*δ*_H_ 1.79)/H-12 (*δ*_H_ 3.04) (Fig. 3). The 7-OH was determined to be *α* configuration by the ROESY cross peak signals of H-15*β* (*δ*_H_ 2.14)/H-7 (*δ*_H_ 4.52) and the coupling constant of H-7 (t, 3.3 Hz) (Fig. 3) [18]. Therefore, the structure of **7** was assigned as ganoaustraldehyde E.

The 1D NMR data of the compounds **8** and **9** (Tables 3 and 2) each showed 26 carbon resonances with a high resemblance to those of compounds **2** and **1**, respectively. Further analysis of the NMR spectroscopic data suggested that **8** and **9** differed from **2** to **1** by the existence of a trisubstituted double bond (C-20, C-22) and a COOH group (C-23), while by the absence of a ketone group (C-20), respectively. These changes suggested that **8** and **9** were two lanostane triterpenes with six carbon degradation (C-24, C-25, C-26, and C-27). The C-23 in **8** and **9** were carboxylic groups which were assigned by the HMBC correlations from H-22 (*δ*_H_ 5.77/5.78 in **8**/**9**) and H_3_-21 (*δ*_H_ 2.17/2.18 in **8**/**9**) to C-23 (*δ*_C_ 171.2/170.3 in **8**/**9**) (Fig. 2), as well as the molecular formulas (C_26_H_34_O_6_ for **8**, C_26_H_34_O_5_ for **9**) which were designated by the HRESIMS analysis. The double bond of C-20(22) were assigned as *E* configuration by the key ROESY correlations between H-22 and H-17 (*δ*_H_ 2.87/2.85 in **8**/**9**), H-16a, H-16b (Fig. 3). Therefore, compounds **8** and **9** were identified as ganoaustrenoic acids A and B (Fig. 1), respectively.

Compound **10** (Fig. 1) returned a protonated ion peak at *m/z* 429.26361 in the HRESIMS analysis, implying it has the molecular formula of C_26_H_36_O_5_ (calcd. for C_26_H_37_O_5_, 429.26410). The molecular formula and the 1D NMR spectroscopic data of **10** (Tables 3 and 2) are reminiscent of those of **9**, implying that **10** was also tetra-nor-lanostane triterpene. The only difference between **10** and **9** is located at C-7, which was indicated by a comparison of the NMR data. The key HMBC correlation from the proton at *δ*_H_ 4.48 (H-7) to C-5 (*δ*_C_ 45.2), C-6 (*δ*_C_ 29.4), C-8 (*δ*_C_ 159.5), C-9 (*δ*_C_ 140.2) (Fig. 2) suggested that C-7 was a hydroxymethine in **10** instead of being a ketone group in **9**. The ROESY correlations of H_3_-18 (*δ*_H_ 0.68)/H-15*β* (*δ*_H_ 2.04)/H-7 (*δ*_H_ 4.48), and the coupling constant of H-7 (t, 3.0 Hz) facilitated to determine the orientation of 7-OH as *α* (Fig. 3). Therefore, compound **10** was identified as ganoaustrenoic acid C.

**Scheme 1:**
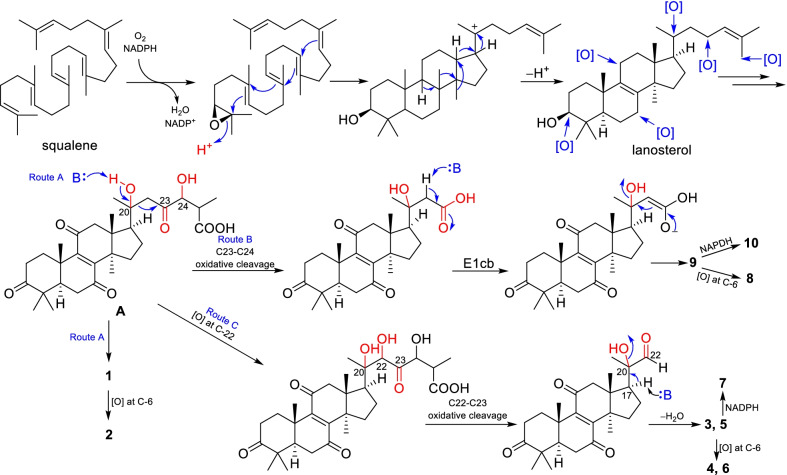
Postulated biosynthetic pathways to compounds **1**–**10**

Since these nor-lanostane are characterized by unusual C-6 oxygenation, and *α*,*β*-aldehyde groups, a biosynthetic proposal is postulated as shown in Scheme 1. The key intermediate lanosterol is biosynthesized from squalene, the common precursor of triterpenes. Further oxygenation on multiple sites of the lanosterol leads to the key intermediate **A** [19, 20]. Abstraction of the proton of OH-20 by the retro-aldol reaction (route A) give compound **1**, which has been further oxygenated at C-6 position to yield **2**. Oxidative cleavage of C23-C24, followed by elimination of the C-20 hydroxy group through E1cb mechanism to produce **9**, which is been further oxygenated or reduced to yield **8** and **10**. Alternatively, **A** undergoes oxygenation at C-22, and oxidative cleavage of C22-C23, and further elimination of C-20 hydroxy group giving compounds **3** and **5**. Late-stage oxygenation and reduction of **3** and **5** produce **4**, **6**, and **7**.

### The anti-NO production activity of the isolates

All the isolates were subjected to screening their inhibition against the NO production in murine monocytic RAW 264.7 macrophages. As a result, only compounds **3** and **4** displayed moderate inhibitory activities with IC_50_ values of 32.5, 34.2 *µ*M, respectively.

## Conclusions

Ten previously undescribed nor-lanostane triterpenes were isolated from the fruiting bodies of *Ganoderma australe*. The chemical structures of the compounds were determined with the aid of extensive NMR and HRESIMS spectroscopic analysis. These compounds are featured by tetra-, penta-, and hexa-nor-lanostane scaffold, and by unusual oxidative modifications at C-6. These findings put the diversity of nor-lanostanes of *Ganoderma* origin one step forward.

## Experimental section

### General experimental procedures

An Autopol IV-T digital polarimeter (Rudolph, Hackettstown, USA) was used to measure the optical rotations. A Shimadzu UV-2401PC UV-vis spectrophotometer (Shimadzu Corporation, Kyoto, Japan) was used to record the UV spectra. The Bruker Ascend 500 MHz, Avance III 600 MHz, or Ascend 800 MHz spectrometers (Bruker Corporation, Karlsruhe, Germany) were used to record the one- and two-dimensional NMR spectra. HRESIMS spectra were measured on a Q Exactive Orbitrap mass spectrometer (Thermo Fisher Scientific, MA, USA). Column chromatography (CC) were run on Sephadex LH-20 (Amersham Biosciences, Uppsala, Sweden) and silica gel (Qingdao Haiyang Chemical Co., Ltd., Qingdao, China). Medium pressure liquid chromatography (MPLC) was performed on an Interchim PuriFlash 450 chromatography system (Interchim Inc., Montlucon Cedex, France). The diameter and length of the column used for MPLC were 14 and 450 mm, respectively. The column was fill with Chromatorex C-18 silica gel (particle size: 40–75 *µ*m, flow rate 40 mL/min, Fuji Silysia Chemical Ltd., Kasugai, Japan). Preparative high performance liquid chromatography (prep.-HPLC) was performed on an Agilent 1260 liquid chromatography system (Agilent Technologies, Santa Clara, CA, USA). The columns used for prep.-HPLC were Agilent Zorbax SB-C18 (5 *µ*m of particle size, i.d. 9.4 mm × length 150 mm, flow rate 7 mL/min), and Agilent Zorbax SB C-8 column (5 *µ*m of particle size, i.d. 9.4 mm × length 250 mm, flow rate 5 mL/min). RPMI 1640 medium (Hyclone, Logan, UT) was used in the anti-nitric oxide production assays. Griess reagent (reagent A and reagent B) was bought from Sigma (Sigma, St. Louis, MO). The plate reader was TECAN Spark 10 M (Tecan Trading AG, Switzerland).

### Fungal material

The fruiting bodies of *Ganoderma australe* were collected in Tongbiguan Natural Reserve, Dehong, Yunnan Province, China, in 2016, and identified by Prof. Yu-Cheng Dai, who is a mushroom research in Institute of Microbiology, Beijing Forestry University. A voucher specimen of *G. australe* was deposited in the Mushroom Bioactive Natural Products Research Group at South-Central Minzu University.

### Extraction and isolation

10 L of mixed solvent CHCl_3_:MeOH (v/v 1:1) was used to extract the constituents from the grounded and dry fruiting bodies of *Ganoderma australe* (3.26 kg) (2.5 L × 4 times) at room temperature. The extract was further resuspended in distilled water and partitioned against ethyl acetate (EtOAc) to afford the EtOAc extract (130 g). The EtOAc extract was fractionated on MPLC by using a stepwise gradient of MeOH in H_2_O (20-100%) to afford seven fractions (A−G).

Fraction D (2.0 g) was separated by Sephadex LH-20 (CHCl_3_:MeOH = 1:1) to afford four subfractions (D1−D4). Subfraction D2 (800 g) was separated by silica gel column chromatography (CC) (petroleum ether-acetone from v/v 15:1 to 1:1) to obtain eleven subfractions (D2a−D2k). Compound **1** (5.2 mg, t_R_ = 12.9 min) was purified from D2k (75 mg) by prep.-HPLC (MeCN-H_2_O: 30−50:50, 30 min, 4 mL/min). Subfraction D4 (610 mg) was subjected to silica gel CC (petroleum ether − acetone from v/v 6:1 to 1:1) and yielded eleven subfractions (D4a − D4k). Subfraction D4d (6.1 mg) was purified by prep.-HPLC (MeCN − H_2_O: 40:60−60:80, 25 min, 4 mL/min) to yield compound **2** (3.8 mg, t_R_ = 16.5 min).

Fraction F (3.6 g) was separated by Sephadex LH-20 (MeOH) to afford twenty-six subfractions (F1-F26). Subfraction F26 (280 mg) was separated by column chromatography (CC) on silica gel (petroleum ether-acetone from v/v 15:1 to 1:1) to obtain four subfractions (F26a-F26d). Subfraction F26a (48 mg) was isolated on prep-HPLC (MeCN − H_2_O: 30:70 − 50:50, 30 min, 4 mL/min) to yield compounds **5** (t_*R*_ = 7.80 min, 1.0 mg), **6** (t_*R*_ = 6.50 min, 0.8 mg), and **4** (t_*R*_ = 11.50 min, 1.3 mg). Compound **3** (0.9 mg, t_R_ = 14.4 min) was purified from F26d (11 mg) by prep.-HPLC (MeCN − H_2_O: 30:70 − 50:50, 30 min, 4 mL/min).

Fraction E (1.6 g) was separated by Sephadex LH-20 (MeOH) to afford twelve subfractions (E1-E12). Subfraction E5 was separated by CC on silica gel (petroleum ether-acetone from v/v 15:1 to 1:1) to obtain four subfractions (E5a-E5d). Compound **8** (2.4 mg, t_*R*_ = 29.4 min) was purified from E5b (23 mg) by prep.-HPLC [MeCN + MeOH (2:1) − H_2_O: 50:50, 40 min, 4 mL/min]. Compound **9** (2.1 mg, t_*R*_ = 20.0 min) was purified from E5c (42 mg) by prep.-HPLC [MeCN + MeOH (2:1) − H_2_O: 40:60–60:40, 25 min, 4 mL/min]. Compound **10** (6.5 mg, t_*R*_ = 13.5 min) was purified from E2e (210 mg) by prep-HPLC (MeCN-H_2_O: 30:70 − 50:50, 25 min, 5 mL/min). Compound **7** (1.8 mg, t_*R*_ = 20.0 min) was purified from E2g (13 mg) by prep.-HPLC (MeCN-H_2_O: 50:50–70:30, 25 min, 4 mL/min).

### Spectroscopic data of compounds

#### Ganoaustratetraenone A (1)

Pale-yellow needles; [*α*]_D_^24^ +164.0 (*c* 0.05, MeOH); UV (MeOH) *λ*_max_ (log *ε*) 255.0 (3.94); ^1^H NMR (600 MHz, CDCl_3_) data, see Table 1, ^13^C NMR (150 MHz, CDCl_3_) data, see Table 2; HRESIMS *m/z* 385.23724 [M + H]^+^, calcd. for C_24_H_33_O_4_, 385.23718.

#### Ganoaustratetraenone B (2)

White powder. [*α*]_D_^24^ +259.4 (*c* 0.09, MeOH); UV (MeOH) *λ*_max_ (log *ε*) 260.0 (3.82); ^1^H NMR (600 MHz, CDCl_3_) data, see Table 1, ^13^C NMR (150 MHz, CDCl_3_) data, see Table 2; HRESIMS *m/z* 401.23224 [M + H]^+^, calcd. for C_24_H_33_O_5_, 401.23280.

#### Ganoaustraldehyde A (3)

White, amorphous powder; [*α*]_D_^24^ +10.9 (*c* 0.05, MeOH); UV (MeOH) *λ*_max_ (log *ε*) 250.0 (3.73); ^1^H NMR (500 MHz, CDCl_3_) data, see Table 1, ^13^C NMR (125 MHz, CDCl_3_) data, see Table 2; HRESIMS *m/z* 397.23721 [M + H]^+^, calcd. for C_25_H_33_O_4_, 397.23734.

#### Ganoaustraldehyde B (4)

White, amorphous powder; [*α*]_D_^24^ +55.8 (*c* 0.05, MeOH); UV (MeOH) *λ*_max_ (log *ε*) 250.0 (3.88); ^1^H NMR (600 MHz, CDCl_3_) data, see Table 1, ^13^C NMR (150 MHz, CDCl_3_) data, see Table 2; HRESIMS *m/z* 413.23221 [M + H]^+^, calcd. for C_25_H_33_O_5_, 413.23225.

#### Ganoaustraldehyde C (5)

White, amorphous powder; [*α*]_D_^24^ +33.5 (*c* 0.05, MeOH); UV (MeOH) *λ*max (log *ε*) 250.0 (4.10); ^1^H NMR (600 MHz, CDCl_3_) data, see Table 1, ^13^C NMR (150 MHz, CDCl_3_) data, see Table 2; HRESIMS *m/z* 397.23743[M + H]^+^, calcd. for C_25_H_33_O_4_, 397.23734.

#### Ganoaustraldehyde D (6)

White, amorphous powder; [*α*]_D_^24^ +68.4 (*c* 0.05, MeOH); UV (MeOH) *λ*_max_ (log *ε*) 250.0 (3.82); ^1^H NMR (600 MHz, CDCl_3_) data, see Table 2, ^13^C NMR (150 MHz, CDCl_3_) data, see Table 2; HRESIMS *m/z* 413.23224[M + H]^+^, calcd. for C_25_H_33_O_5_, 413.23225.

#### Ganoaustraldehyde E (7)

Pale-yellow oil. [*α*]_D_^24^ +156.8 (*c* 0.09, MeOH); UV (MeOH) *λ*_max_ (log *ε*) 250.0 (4.62); ^1^H NMR (600 MHz, CDCl_3_) data, see Table 2, ^13^C NMR (150 MHz, CDCl_3_) data, see Table 2; HRESIMS *m/z* 399.25305 [M + H]^+^, calcd. for C_25_H_35_O_4_, 399.25353.

#### Ganoaustrenoic acid A (8)

White powder. [*α*]_D_^24^ +162.7 (*c* 0.06, MeOH); UV (MeOH) *λ*_max_ (log *ε*) 220.0 (4.08); ^1^H NMR (600 MHz, CDCl_3_) data, see Table 2, ^13^C NMR (150 MHz, CDCl_3_) data, see Table 2; HRESIMS *m/z* 443.24299 [M + H]^+^, calcd. for C_26_H_35_O_6_, 443.24336.

#### Ganoaustrenoic acid B (9)

White powder. [*α*]_D_^24^ +10.3 (*c* 0.05, MeOH); UV (MeOH) *λ*_max_ (log *ε*) 225.0 (4.23); ^1^H NMR (600 MHz, CDCl_3_) data, see Table 2, ^13^C NMR (150 MHz, CDCl_3_) data, see Table 2; HRESIMS *m/z* 427.24808 [M + H]^+^, calcd. for C_26_H_35_O_5_, 427.24845.

#### 10 ganoaustrenoic acid C (10)

Yellowish oil. [*α*]_D_^24^ +84.3 (*c* 0.07, MeOH); UV (MeOH) *λ*_max_ (log *ε*) 225.0 (3.90); ^1^H NMR (600 MHz, CDCl_3_) data, see Table 2, ^13^C NMR (200 MHz, CDCl_3_) data, see Table 2; HRESIMS *m/z* 429.26361 [M + H]^+^, calcd. for C_26_H_37_O_5_, 429.26410.

### Nitric oxide production in RAW 264.7 macrophages

The murine monocytic RAW 264.7 macrophages were cultured in RPMI 1640 medium 10% Fetal Bovine Serum (FBS). Firstly, the DMSO stocks of the compounds were made, and further been serially diluted in media to make different concentrations of stocks. The cell mixtures were distributed into 96-well plates (2 × 10^5^ cells/well) and preincubated in a humid atmosphere with 5% CO_2_ for 24 h at 37 °C. Then different concentrations of stocks of the test compounds were added into each well, the maximum concentration of the test compound was 25 *µ*M. Then the lipopolysaccharides (LPS) were added to each cell (final concentration 1 *µ*g/mL), and continued to incubate for another 18 h. After adding 100 *µ*L of Griess reagent to 100 *µ*L of each supernatant from the LPS-treated or LPS- and compound-treated cells in triplicates, and incubated for another 5 min, the nitric oxide (NO) production of each cell was assessed by measuring the absorbance at 570 nm. Pyrrolidine dithiocarbamate (PDTC) was used as the positive control (IC_50_ 20.0 *µ*M).

## Supplementary Information

**Additional file 1**. The NMR, HRESIMS spectra of compounds **1**–**10**
