# Supplementary material for: Tetra-, penta-, and hexa-nor-lanostane triterpenes from the medicinal fungus Ganoderma australe
Source: Nat Prod Bioprospect. 2022 Aug 16;12(1):32. doi: 10.1007/s13659-022-00356-x (PMC9378796; doi:10.1007/s13659-022-00356-x)

Additional file 1 for

**Tetra-, Penta-, and Hexa-Nor-Lanostane Triterpenes from the Medicinal Fungus *Ganoderma australe***

Lin Zhou^1^, Subiy Akbar^1^, Meng-Xi Wang, He-Ping Chen*, Ji-Kai Liu*

*School of Pharmaceutical Sciences, South-Central Minzu University, Wuhan 430074, China*

Corresponding authors:

Dr. H.-P. Chen, email: [chenhp@mail.scuec.edu.cn](mailto:chenhp@mail.scuec.edu.cn)

Prof. Dr. J.-K. Liu, email: [liujikai@mail.scuec.edu.cn](mailto:liujikai@mail.scuec.edu.cn)

^1^These authors contribute equally to this work.

**Contents**

[_Toc9865](#_Toc9865)

[Figure S1. ^1^H NMR spectrum of **1** (600 MHz, CDCl_3_). 3](#_Toc14673)

[Figure S2. ^13^C NMR and DEPT spectra of **1** (150 MHz, CDCl_3_). 4](#_Toc27216)

[Figure S3. HSQC spectrum of **1**. 5](#_Toc3455)

[Figure S4. ^1^H-^1^H COSY spectrum of **1**. 6](#_Toc27360)

[Figure S5. HMBC spectrum of **1**. 7](#_Toc421)

[Figure S6. ROESY spectrum of **1**. 8](#_Toc25997)

[Figure S7. HRESIMS spectrum of **1**. 9](#_Toc25184)

[Figure S8. ^1^H NMR spectrum of **2** (600 MHz, CDCl_3_) 10](#_Toc20147)

[Figure S9. ^13^C and DEPT NMR spectra of **2** (150 MHz, CDCl_3_) 11](#_Toc3080)

[Figure S10. HSQC spectrum of **2**. 12](#_Toc3340)

[Figure S11. HMBC spectrum of **2**. 13](#_Toc24362)

[Figure S12. ^1^H-^1^H COSY spectrum of **2**. 14](#_Toc10436)

[Figure S13. ROESY spectrum of **2**. 15](#_Toc3528)

[Figure S14. HRESIMS of **2**. 16](#_Toc21558)

[Figure S15. ^1^H NMR spectrum of **3** (500 MHz, CD_3_OD). 17](#_Toc12206)

[Figure S16. ^13^C NMR and DEPT spectra of **3** (125 MHz, CD_3_OD). 18](#_Toc9070)

[Figure S17. Enlarged HSQC spectrum (0.8-3.7 ppm) of **3**. 19](#_Toc15967)

[Figure S18. Enlarged HSQC spectrum (9.8-10.2 ppm) of **3**. 20](#_Toc12523)

[Figure S19. ^1^H-^1^H COSY spectrum of **3**. 21](#_Toc23903)

[Figure S20. HMBC spectrum of **3**. 22](#_Toc8495)

[Figure S21. ROESY spectrum of **3**. 23](#_Toc14827)

[Figure S22. HRESIMS spectrum of **3**. 24](#_Toc17444)

[Figure S23. ^1^H NMR spectrum of **4** (600 MHz, CDCl_3_). 25](#_Toc25953)

[Figure S24. ^13^C NMR and DEPT90 spectra of **4** (150 MHz, CDCl_3_). 26](#_Toc19043)

[Figure S25. Enlarged HSQC spectrum (1.0-4.8) of **4**. 27](#_Toc26590)

[Figure S26. Enlarged HSQC spectrum (9.7-10.3) of **4**. 28](#_Toc30166)

[Figure S27. ^1^H-^1^H COSY spectrum of **4**. 29](#_Toc29922)

[Figure S28. HMBC spectrum of **4**. 30](#_Toc10322)

[Figure S29. ROESY spectrum of **4**. 31](#_Toc14839)

[Figure S30. HRESIMS report of **4**. 32](#_Toc2074)

[Figure S31. ^1^H NMR spectrum of **5** (600 MHz, CDCl_3_). 33](#_Toc16301)

[Figure S32. ^13^C NMR and DEPT spectra of **5** (150 MHz, CDCl_3_). 34](#_Toc29037)

[Figure S33. Enlarged HSQC spectrum (0.8-3.7) of **5**. 35](#_Toc22385)

[Figure S34. Enlarged HSQC spectrum (9.8-10.2) of **5**. 36](#_Toc25996)

[Figure S35. ^1^H-^1^H COSY spectrum of **5**. 37](#_Toc9468)

[Figure S36. HMBC spectrum of **5**. 38](#_Toc27349)

[Figure S37. ROESY spectrum of **5**. 39](#_Toc2170)

[Figure S38. HRESIMS report of **5**. 40](#_Toc12016)

[Figure S39. ^1^H NMR spectrum of **6** (600 MHz, CDCl_3_). 41](#_Toc5774)

[Figure S40. ^13^C NMR and DEPT spectra of **6** (150 MHz, CDCl_3_). 42](#_Toc797)

[Figure S41. Enlarged HSQC spectrum (1.0-4.5) of **6**. 43](#_Toc30182)

[Figure S42. Enlarged HSQC spectrum (9.8-10.1) of **6**. 44](#_Toc19484)

[Figure S43. ^1^H-^1^H COSY spectrum of **6**. 45](#_Toc30202)

[Figure S44. HMBC spectrum of **6**. 46](#_Toc2641)

[Figure S45. ROESY spectrum of **6**. 47](#_Toc4389)

[Figure S46. HRESIMS report of **6**. 48](#_Toc1042)

[Figure S47. ^1^H NMR spectrum of **7** (600 MHz, CDCl_3_) 49](#_Toc23619)

[Figure S48. ^13^C and DEPT NMR spectra of **7** (150 MHz, CDCl_3_) 50](#_Toc11063)

[Figure S49. HSQC spectrum of **7**. 51](#_Toc14450)

[Figure S50. HMBC spectrum of **7**. 52](#_Toc24684)

[Figure S51. ^1^H-^1^H COSY spectrum of **7**. 53](#_Toc32684)

[Figure S52. ROESY spectrum of **7**. 54](#_Toc2516)

[Figure S53. HRESIMS of **7**. 55](#_Toc27470)

[Figure S54. ^1^H NMR spectrum of **8** (600 MHz, CDCl_3_) 56](#_Toc24573)

[Figure S55. ^13^C and DEPT NMR spectra of **8** (150 MHz, CDCl_3_) 57](#_Toc11214)

[Figure S56. HSQC spectrum of **8**. 58](#_Toc11058)

[Figure S57. HMBC spectrum of **8**. 59](#_Toc9005)

[Figure S58. ^1^H-^1^H COSY spectrum of **8**. 60](#_Toc15541)

[Figure S59. ROESY spectrum of **8**. 61](#_Toc32743)

[Figure S60. HRESIMS of **8**. 62](#_Toc2657)

[Figure S61. ^1^H NMR spectrum of **9** (600 MHz, CDCl_3_) 63](#_Toc9327)

[Figure S62. ^13^C and DEPT NMR spectra of **9** (150 MHz, CDCl_3_) 64](#_Toc18629)

[Figure S63. HSQC spectrum of **9**. 65](#_Toc26485)

[Figure S64. HMBC spectrum of **9**. 66](#_Toc32444)

[Figure S65. ^1^H-^1^H COSY spectrum of **9**. 67](#_Toc23441)

[Figure S66. ROESY spectrum of **9**. 68](#_Toc9988)

[Figure S67. HRESIMS of **9**. 69](#_Toc260)

[Figure S68. ^1^H NMR spectrum of **10** (600 MHz, CDCl_3_) 70](#_Toc22381)

[Figure S69. ^13^C NMR spectrum of **10** (200 MHz, CDCl_3_) 71](#_Toc6821)

[Figure S70. HSQC spectrum of **10**. 72](#_Toc25233)

[Figure S71. HMBC spectrum of **10**. 73](#_Toc26436)

[Figure S72. ^1^H-^1^H COSY spectrum of **10**. 74](#_Toc20532)

[Figure S73. ROESY spectrum of **10**. 75](#_Toc19199)

[Figure S74. HRESIMS of **10**. 76](#_Toc10682)

## Figure S1. ^1^H NMR spectrum of **1** (600 MHz, CDCl_3_).


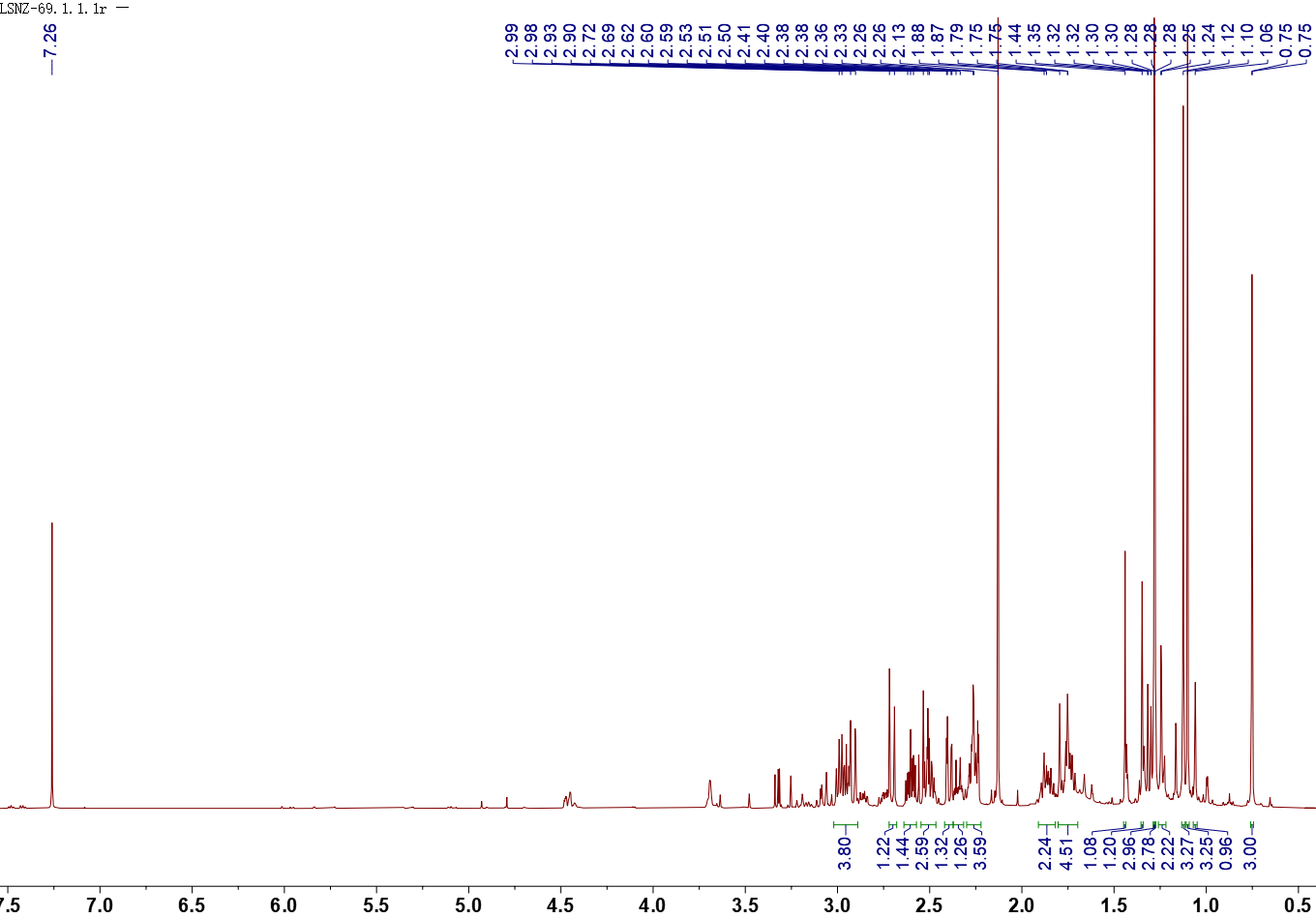


## Figure S2. ^13^C NMR and DEPT spectra of **1** (150 MHz, CDCl_3_).

## Figure S3. HSQC spectrum of **1**.


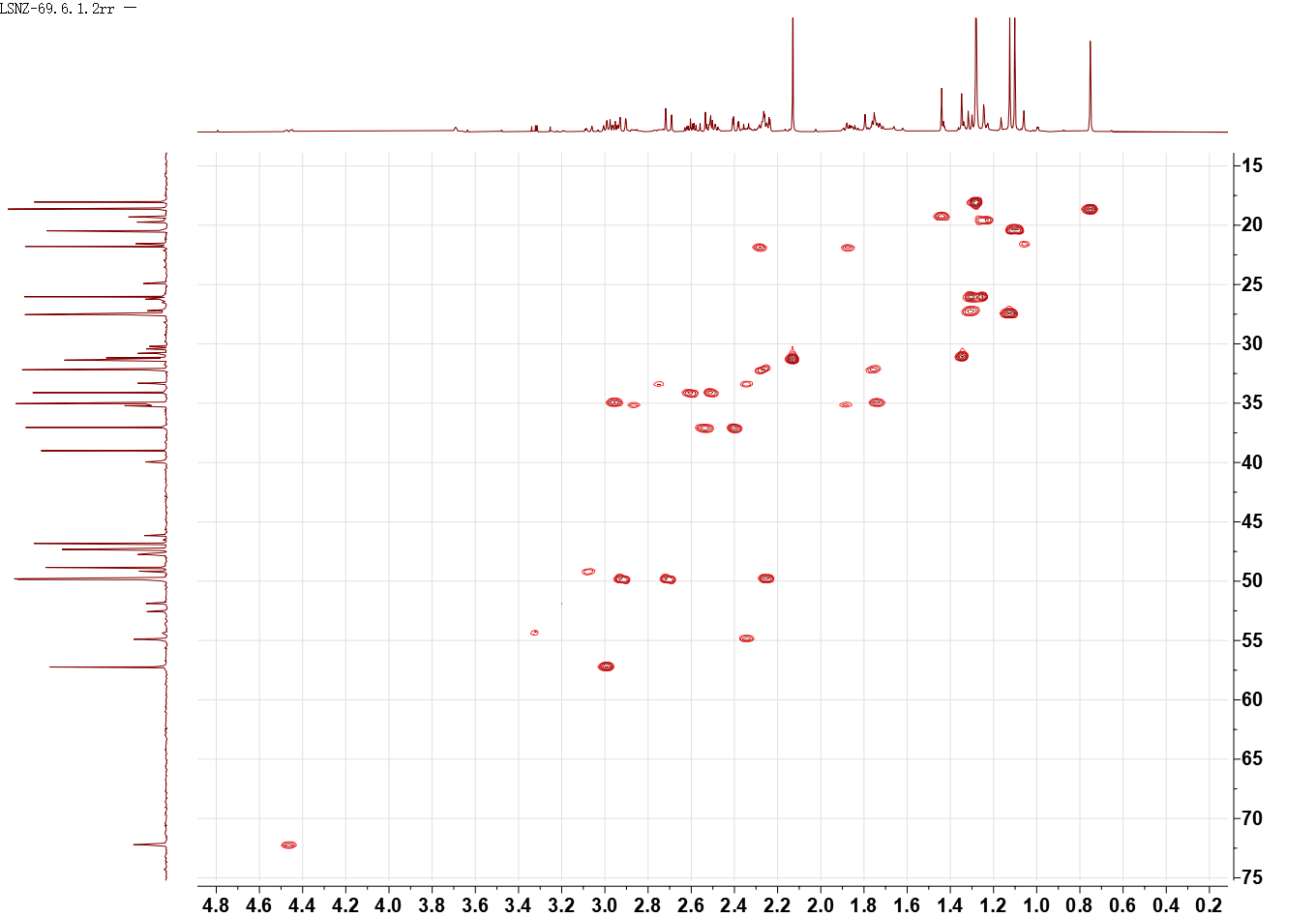


## Figure S4. ^1^H-^1^H COSY spectrum of **1**.


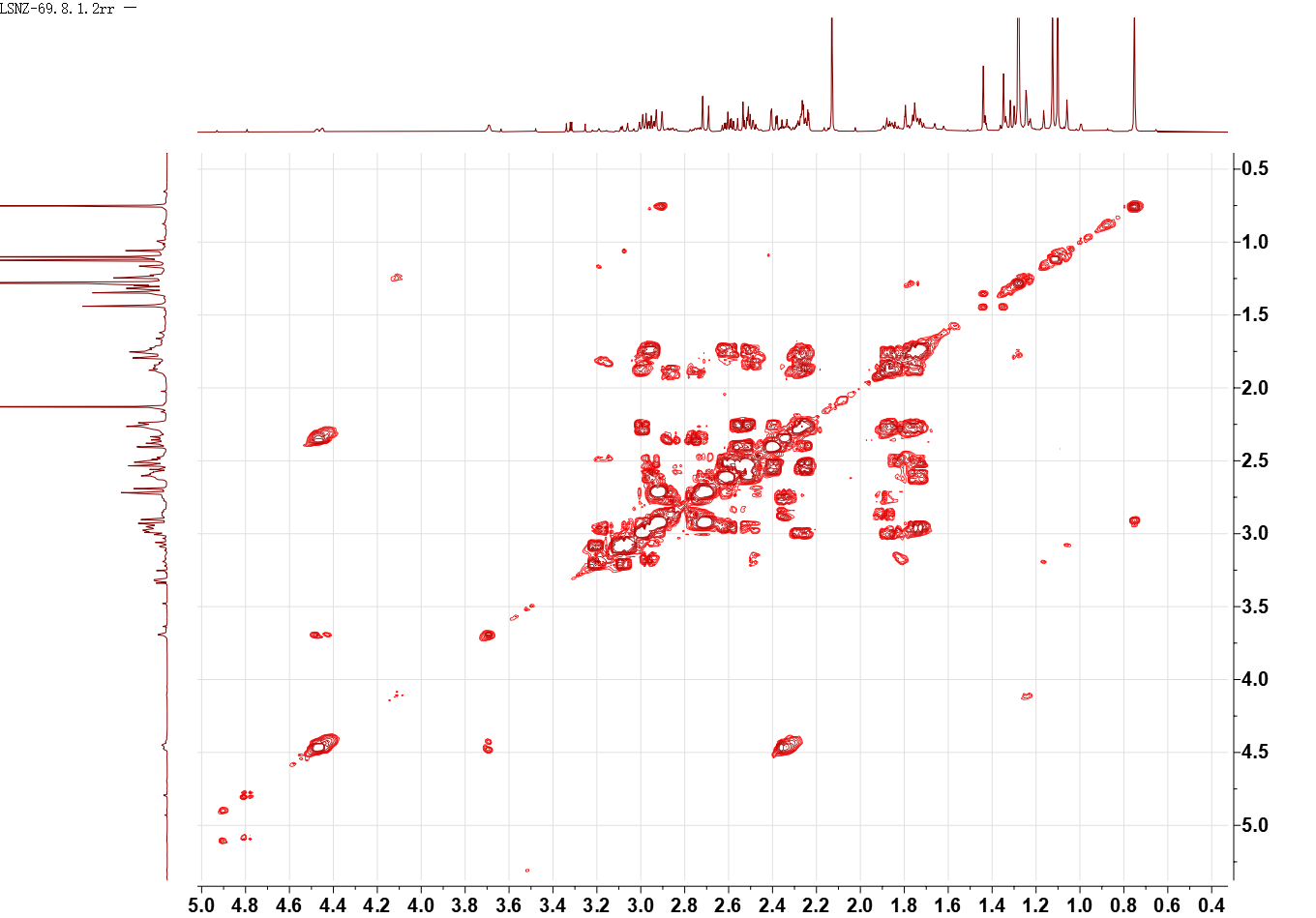


## Figure S5. HMBC spectrum of **1**.


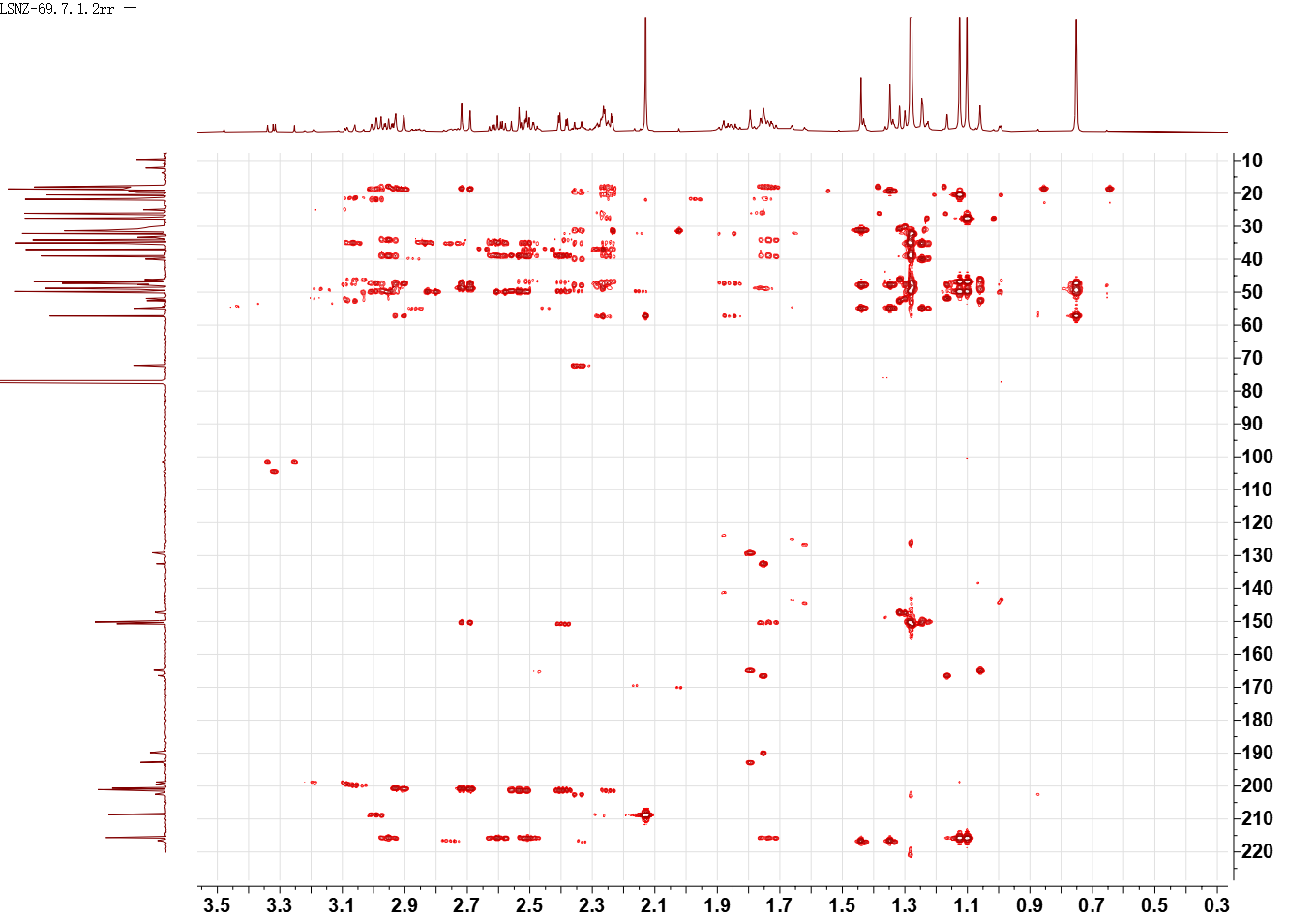


## Figure S6. ROESY spectrum of **1**.


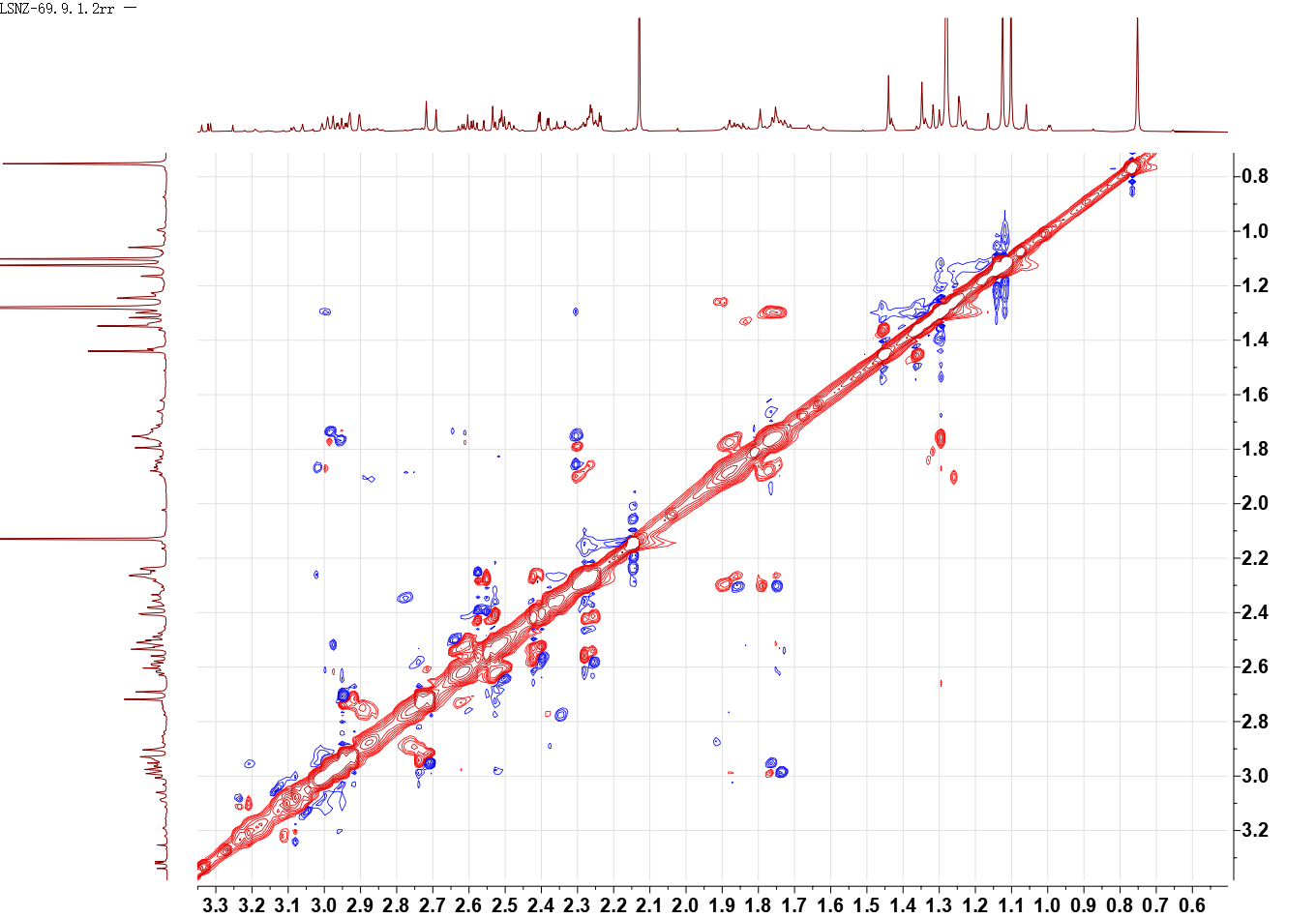


## Figure S7. HRESIMS spectrum of **1**.


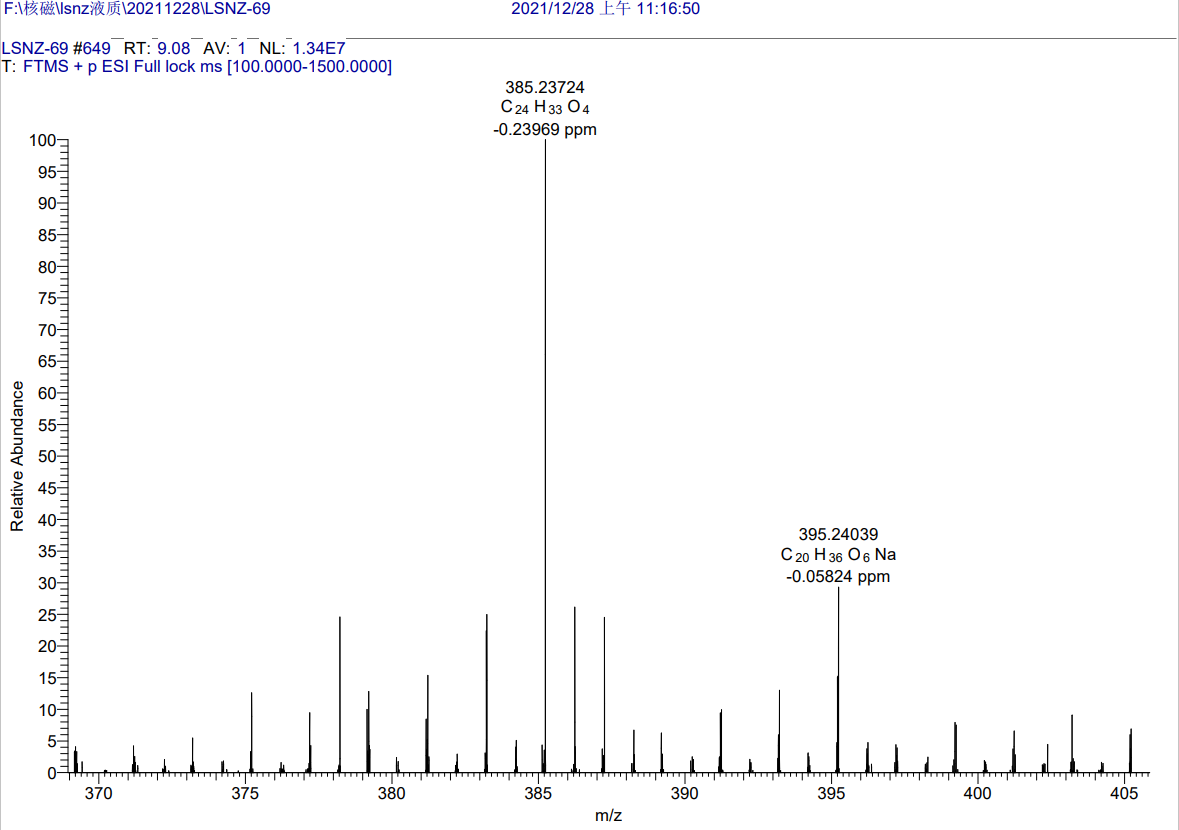


## Figure S8. ^1^H NMR spectrum of **2** (600 MHz, CDCl_3_)

## Figure S9. ^13^C and DEPT NMR spectra of **2** (150 MHz, CDCl_3_)

## Figure S10. HSQC spectrum of **2**.

## Figure S11. HMBC spectrum of **2**.

## Figure S12. ^1^H-^1^H COSY spectrum of **2**.

## Figure S13. ROESY spectrum of **2**.

## Figure S14. HRESIMS of **2**.


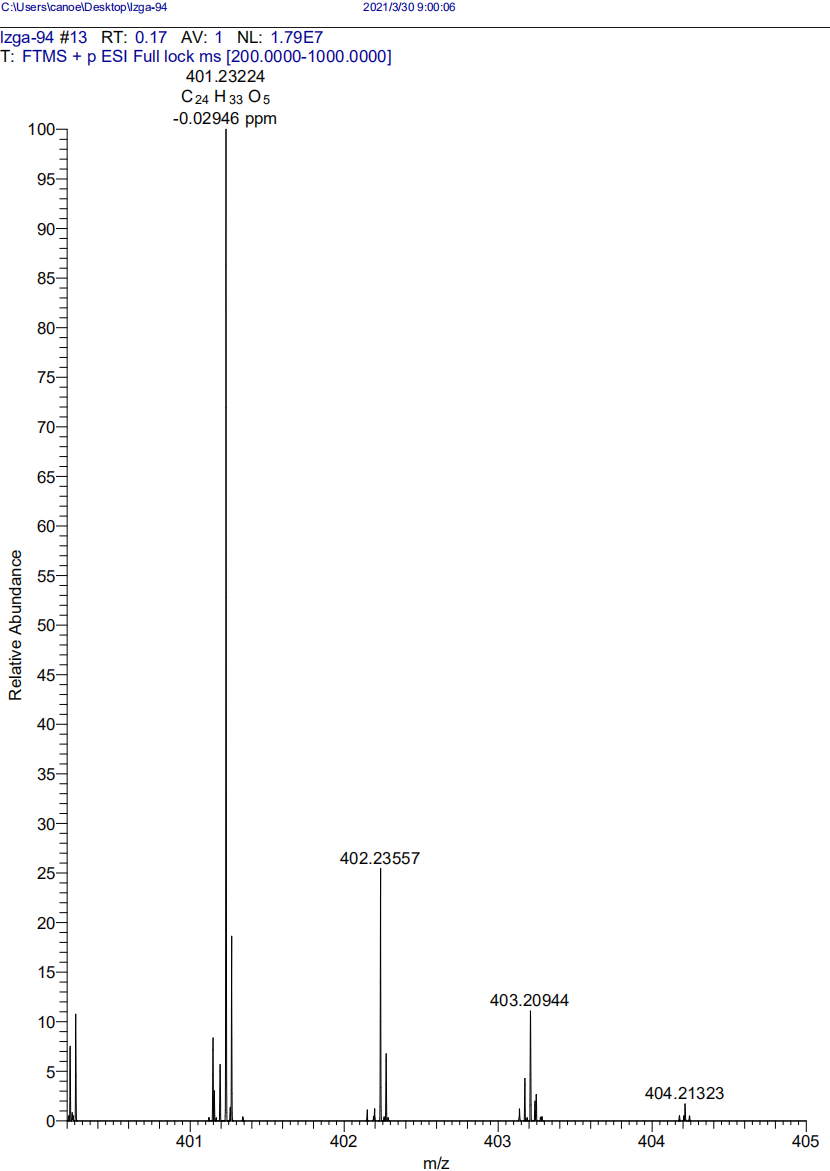


## Figure S15. ^1^H NMR spectrum of **3** (500 MHz, CD_3_OD).


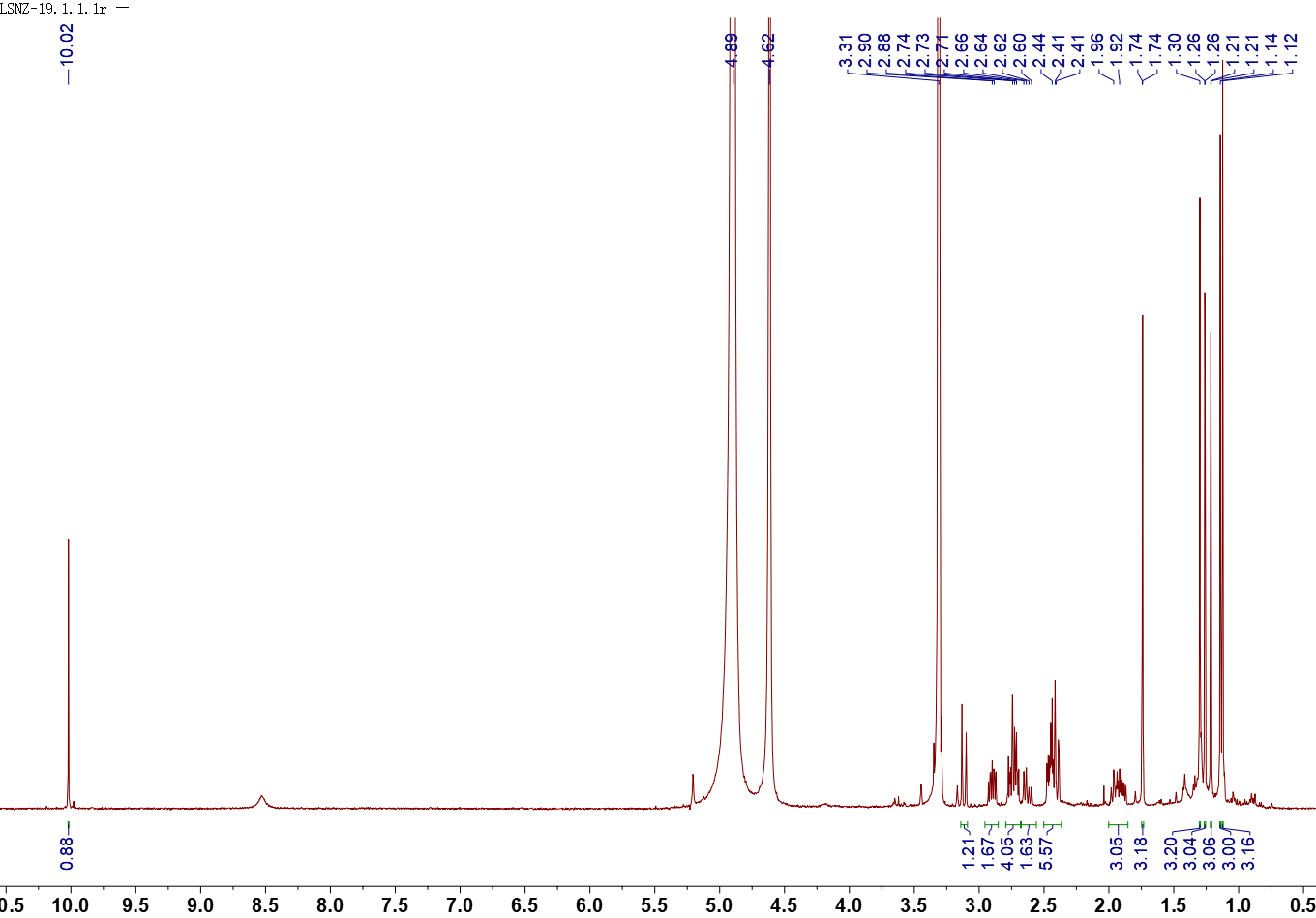


## Figure S16. ^13^C NMR and DEPT spectra of **3** (125 MHz, CD_3_OD).

## Figure S17. Enlarged HSQC spectrum (0.8-3.7 ppm) of **3**.


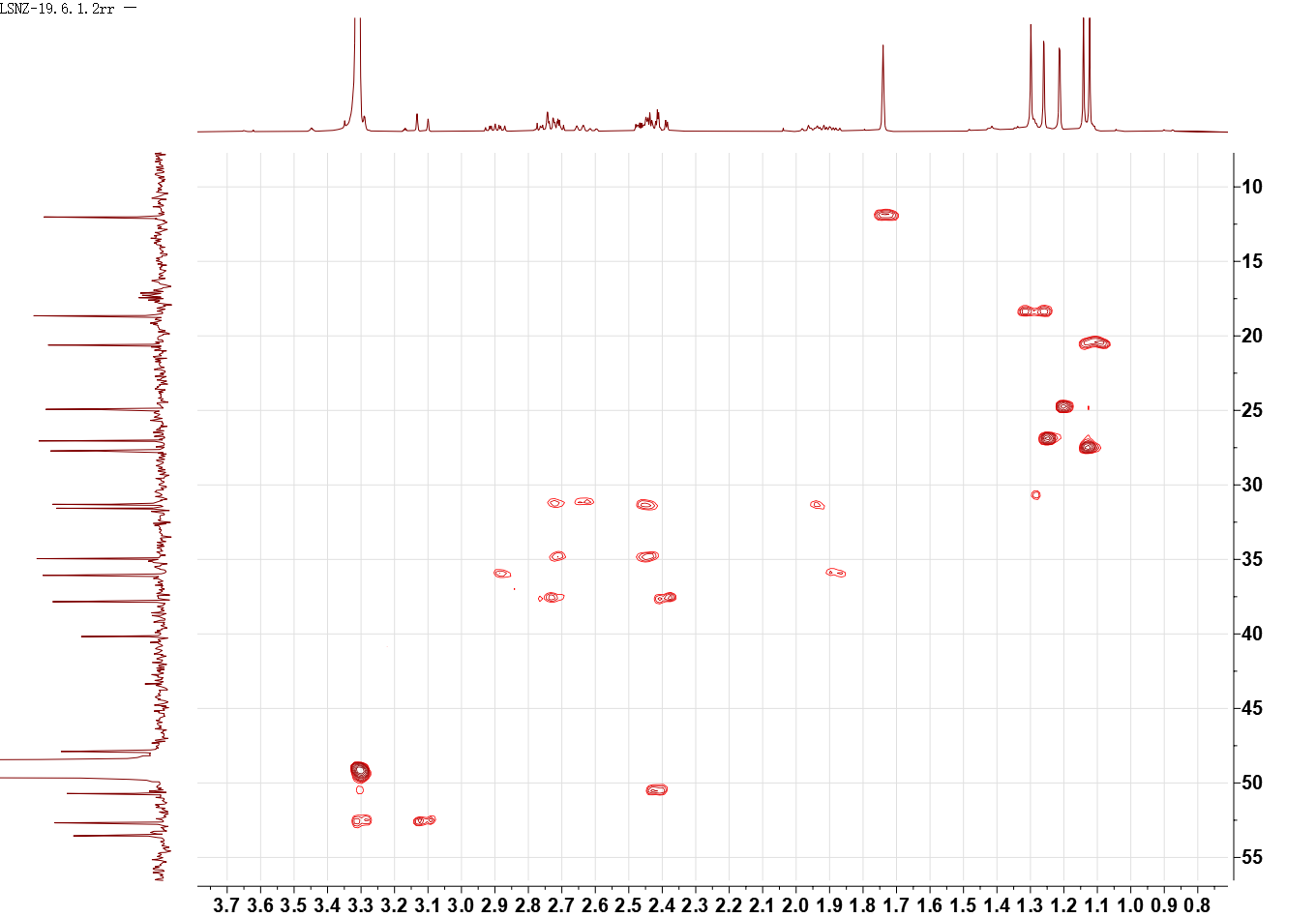


## Figure S18. Enlarged HSQC spectrum (9.8-10.2 ppm) of **3**.


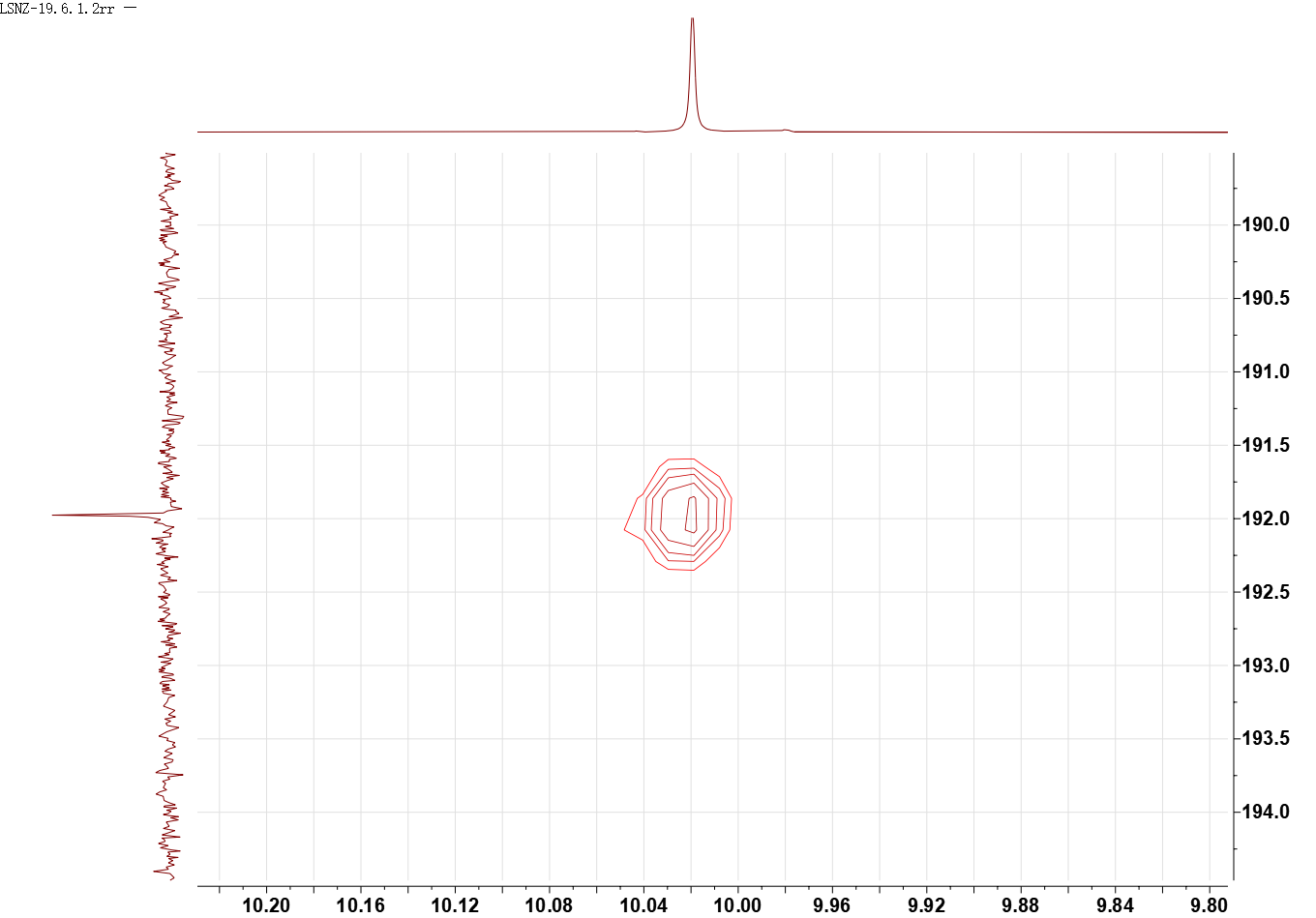


## Figure S19. ^1^H-^1^H COSY spectrum of **3**.


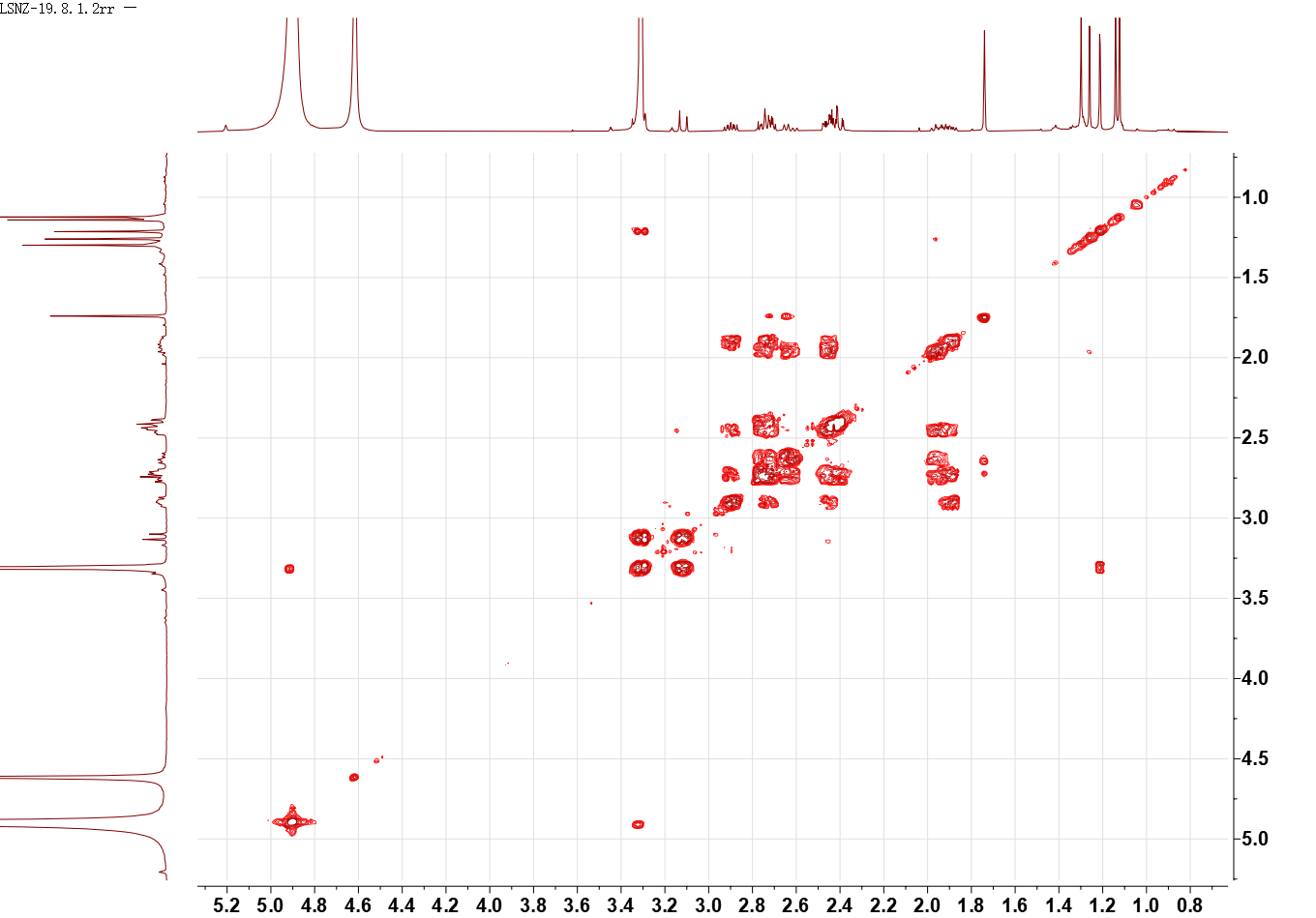


## Figure S20. HMBC spectrum of **3**.


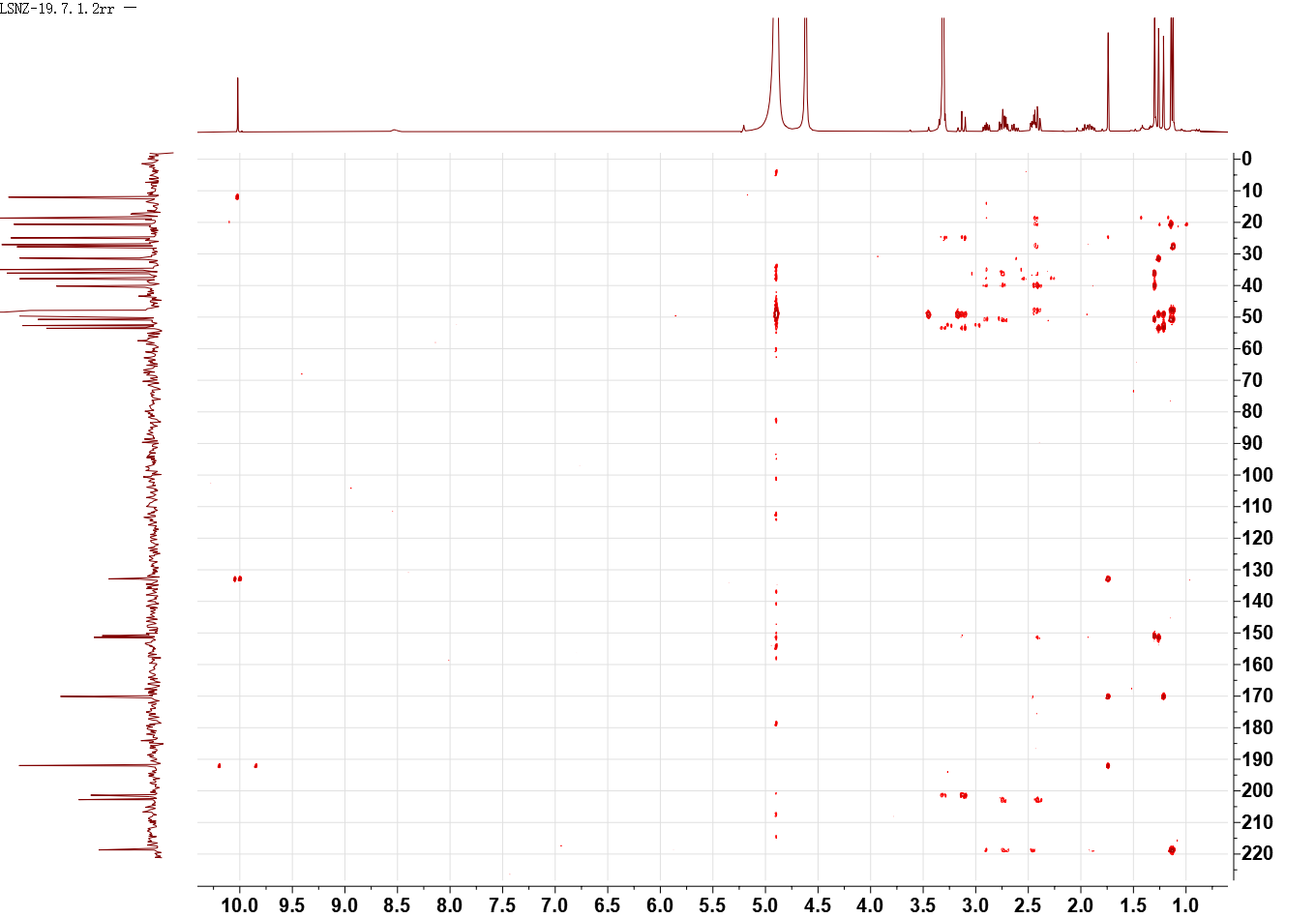


## Figure S21. ROESY spectrum of **3**.

## Figure S22. HRESIMS spectrum of **3.**


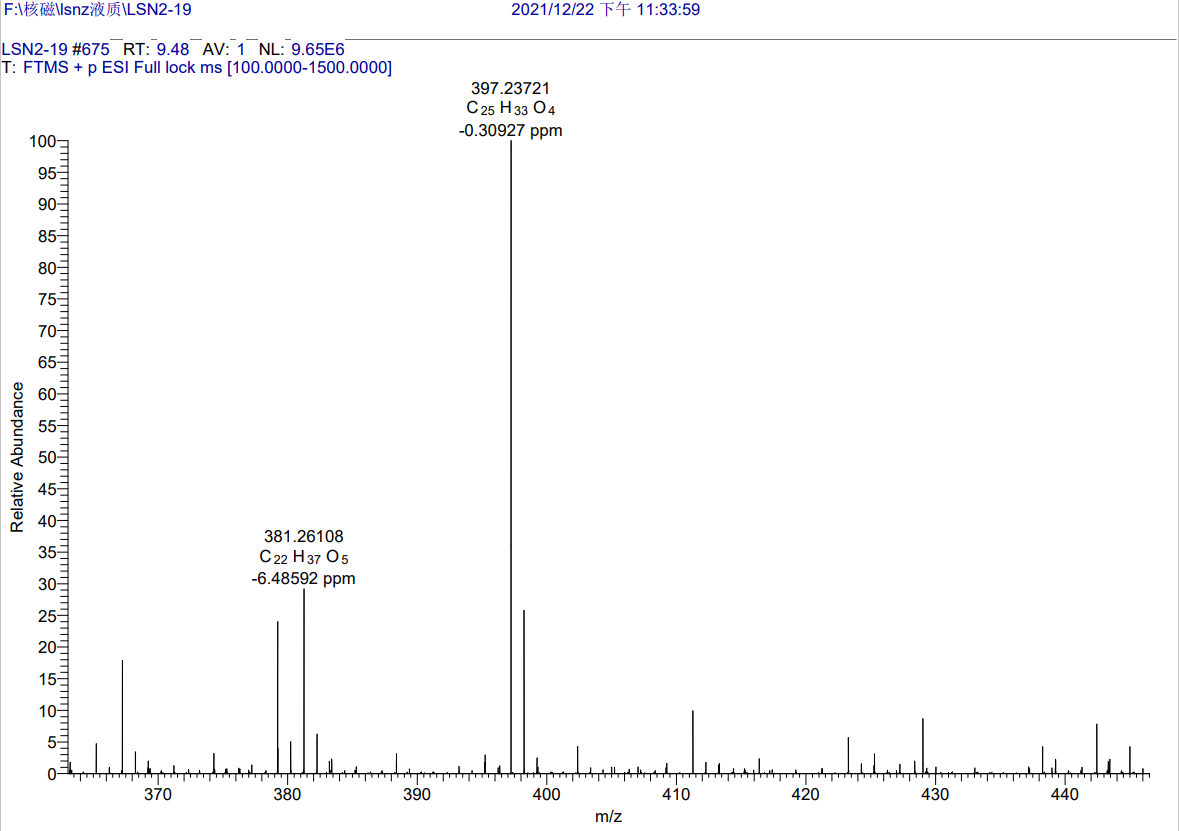


## Figure S23. ^1^H NMR spectrum of **4** (600 MHz, CDCl_3_).


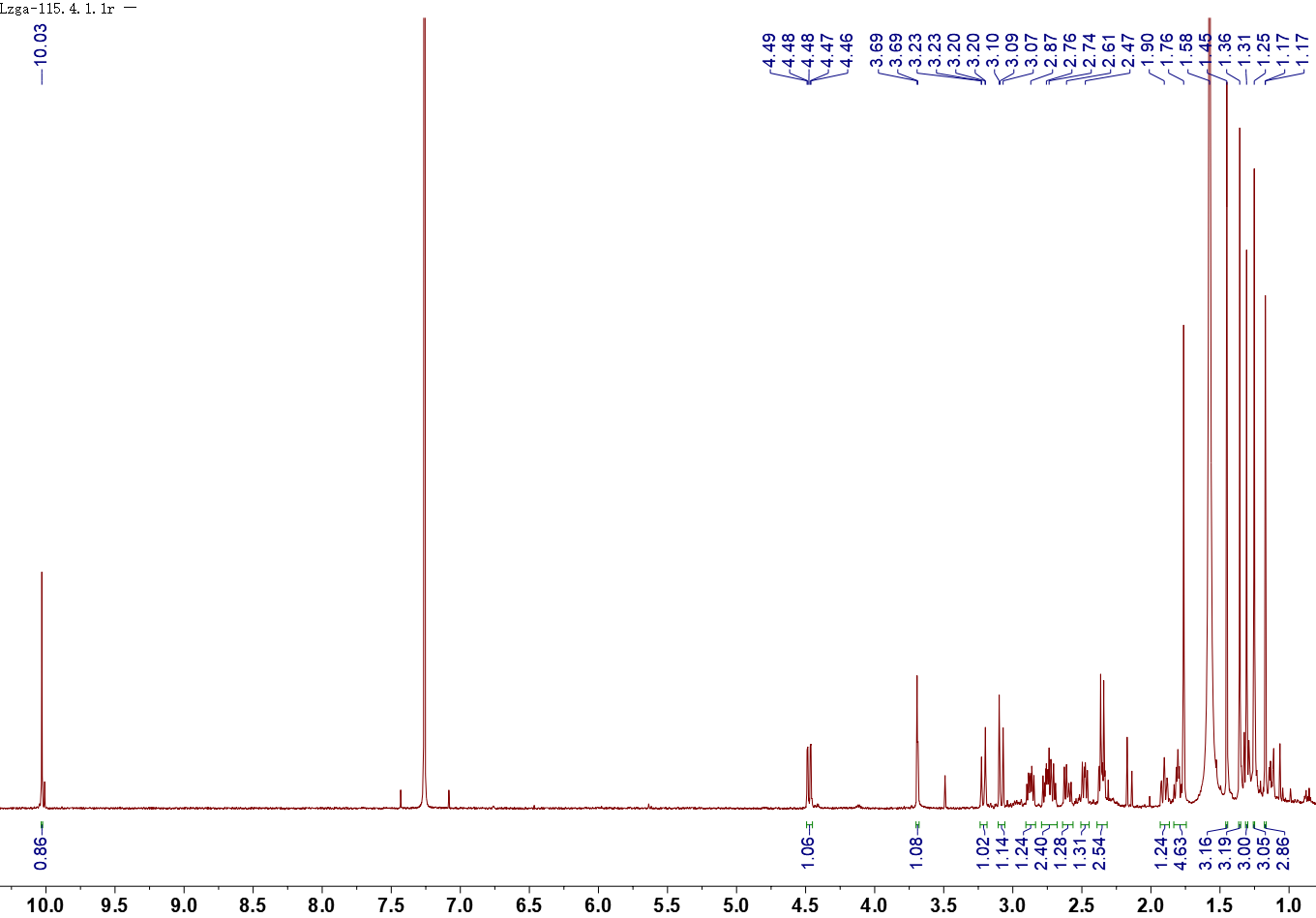


## Figure S24. ^13^C NMR and DEPT90 spectra of **4** (150 MHz, CDCl_3_).

## Figure S25. Enlarged HSQC spectrum (1.0-4.8) of **4**.


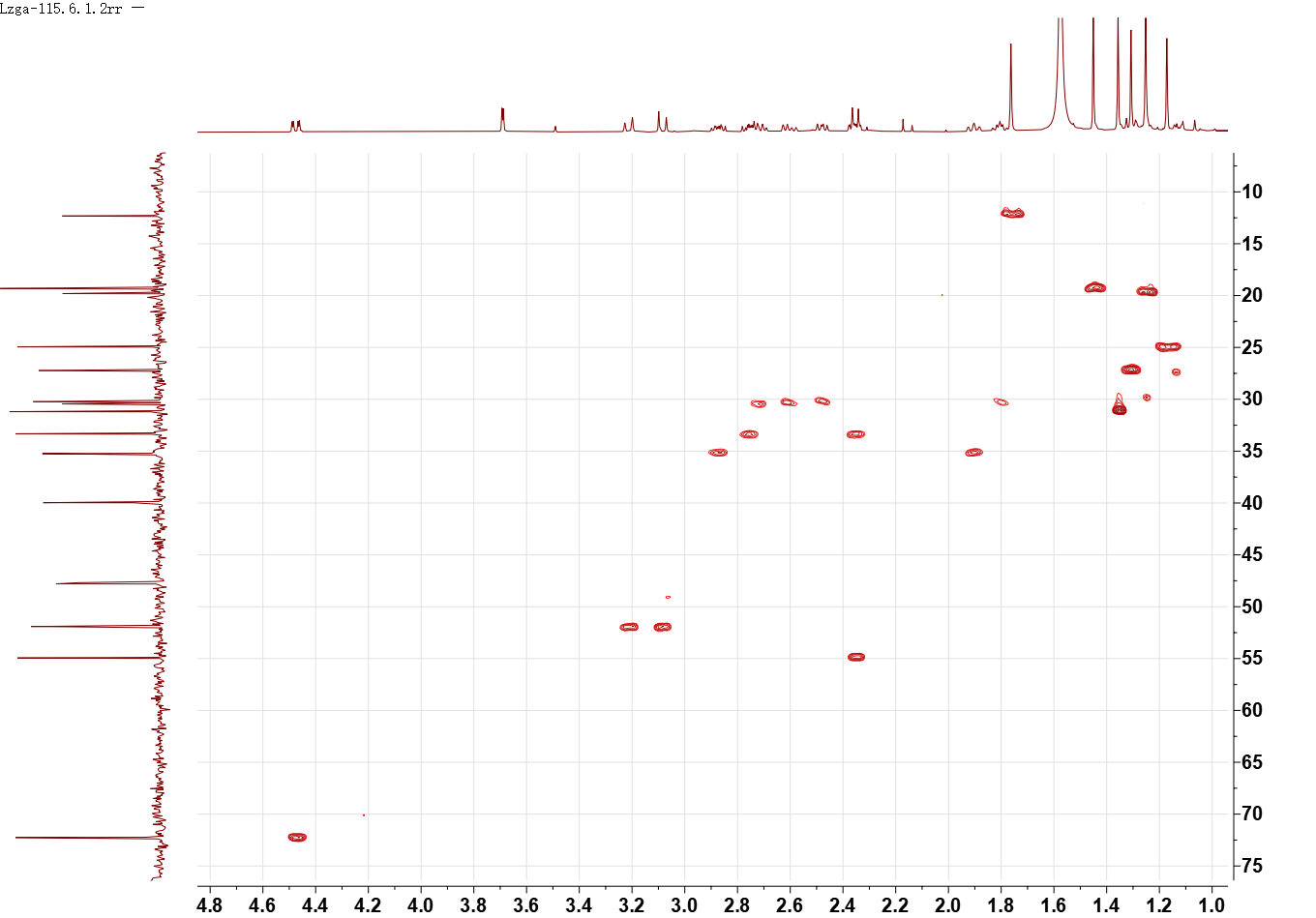


## Figure S26. Enlarged HSQC spectrum (9.7-10.3) of **4**.


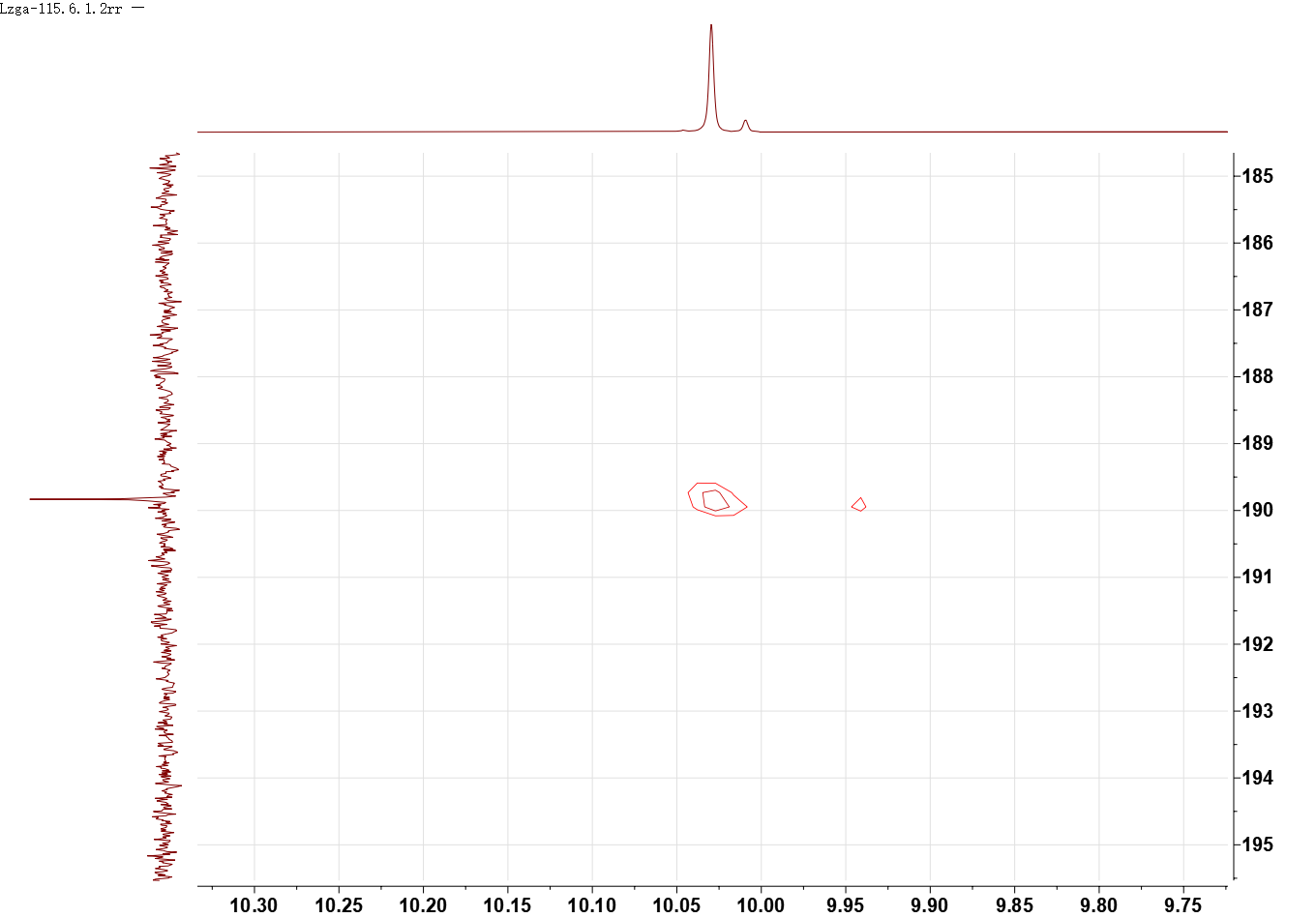


## Figure S27. ^1^H-^1^H COSY spectrum of **4**.


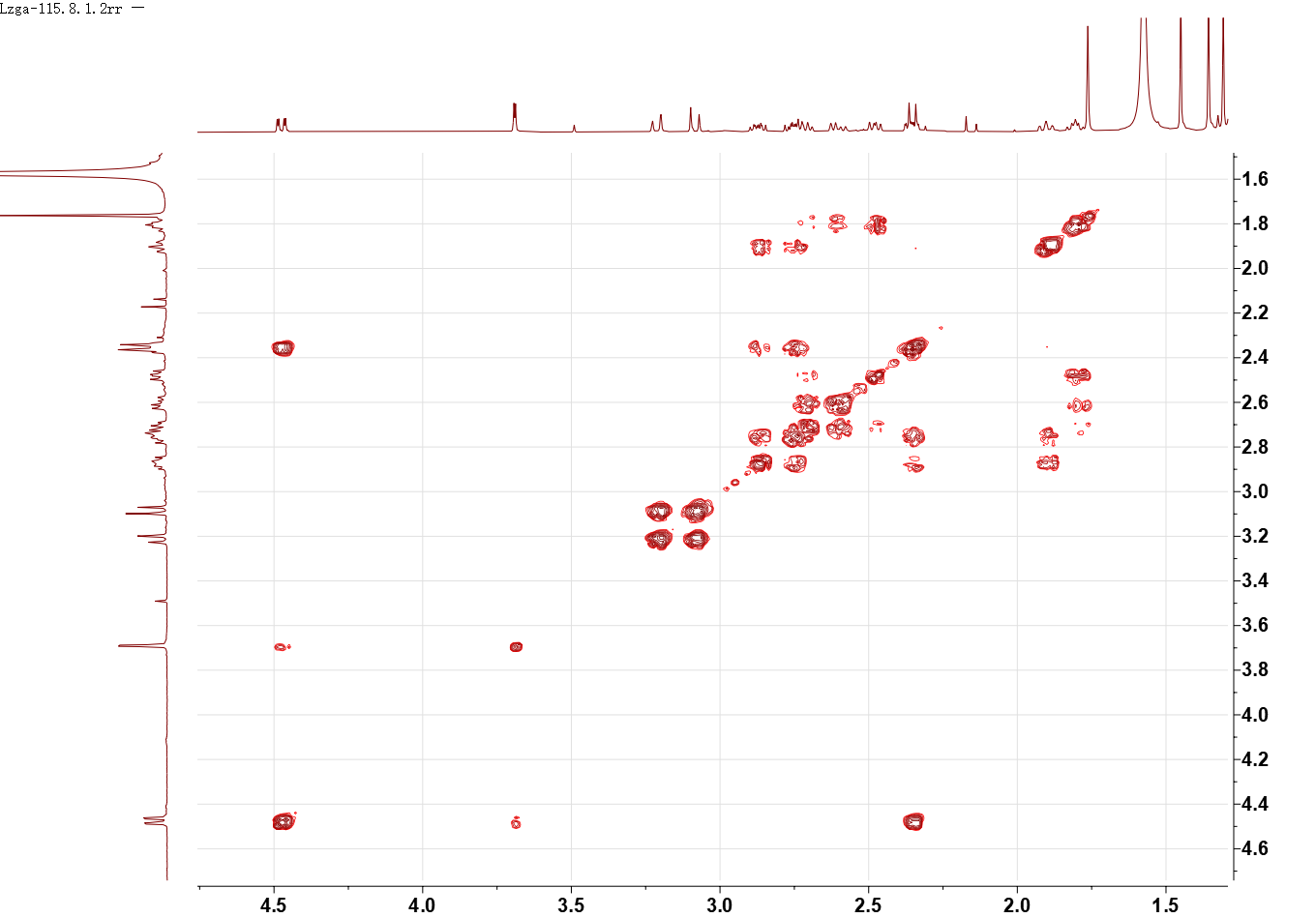


## Figure S28. HMBC spectrum of **4**.


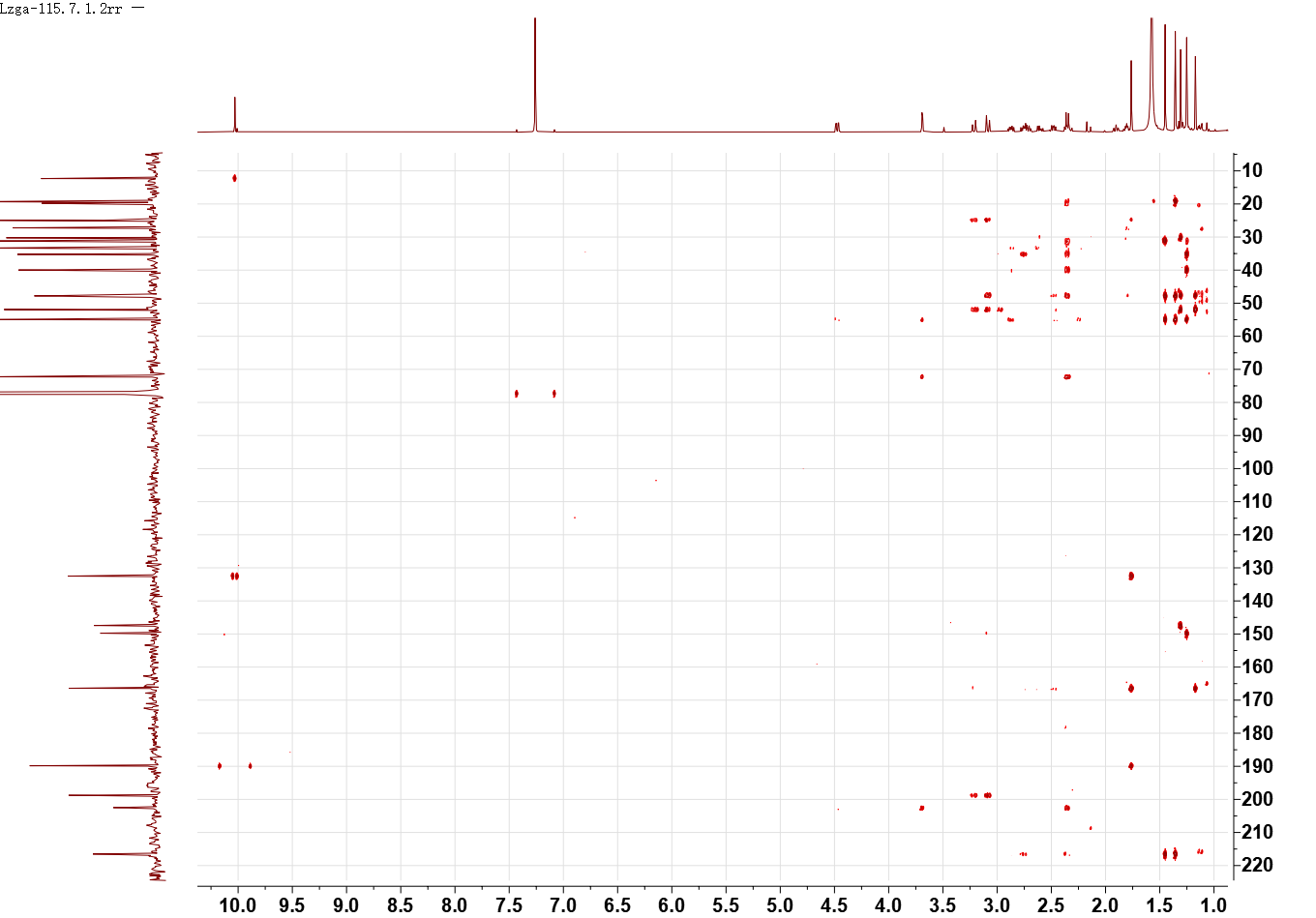


## Figure S29. ROESY spectrum of **4**.

## Figure S30. HRESIMS report of **4**.


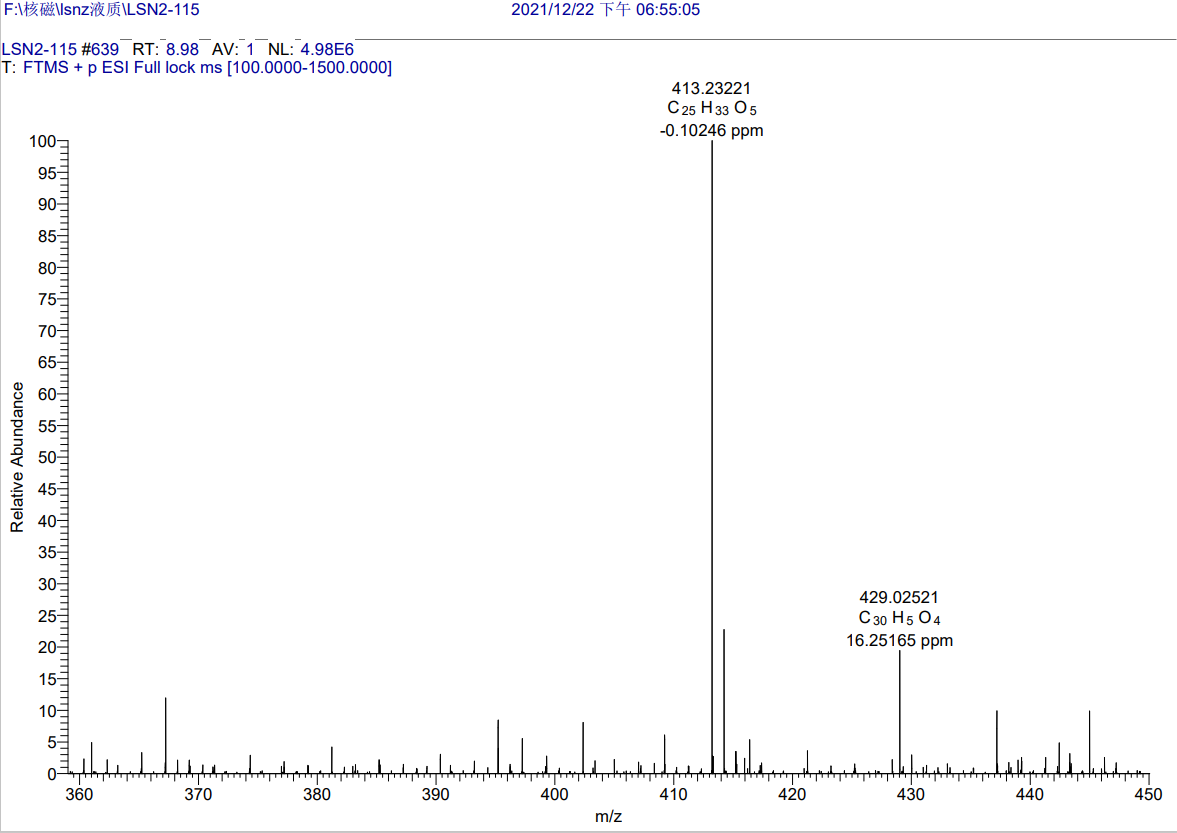


## Figure S31. ^1^H NMR spectrum of **5** (600 MHz, CDCl_3_).


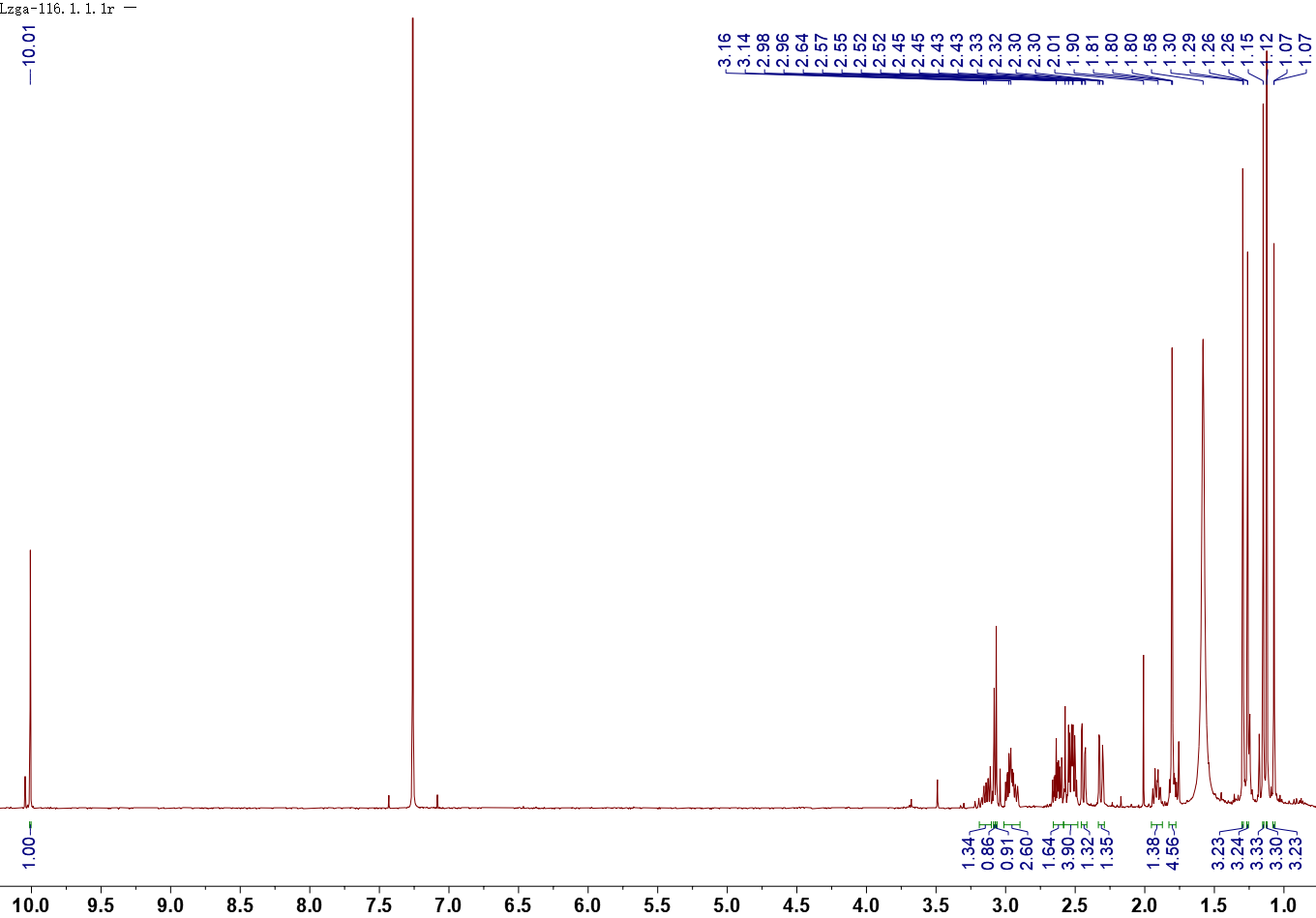


## Figure S32. ^13^C NMR and DEPT spectra of **5** (150 MHz, CDCl_3_).

## Figure S33. Enlarged HSQC spectrum (0.8-3.7) of **5**.


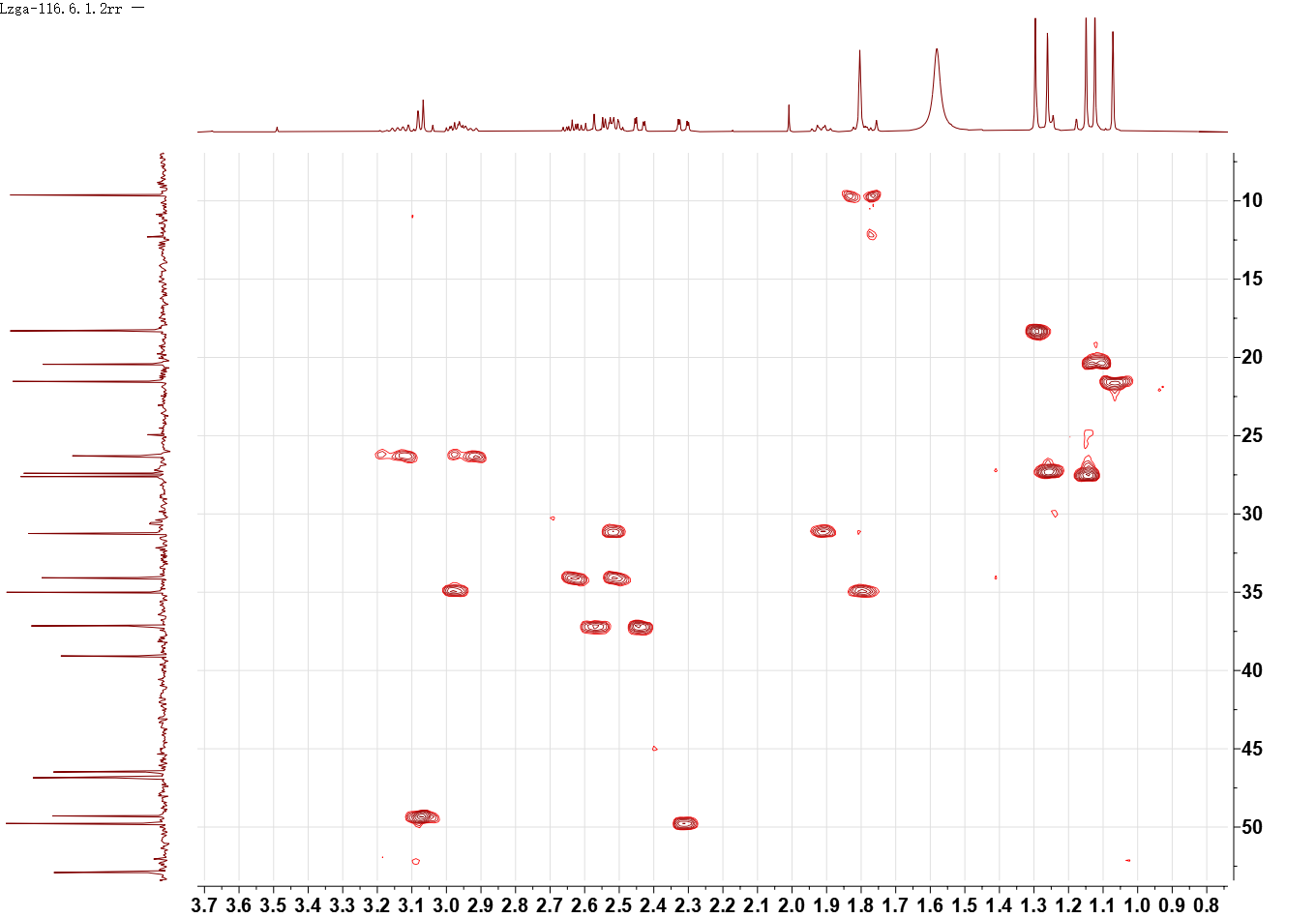


## Figure S34. Enlarged HSQC spectrum (9.8-10.2) of **5**.


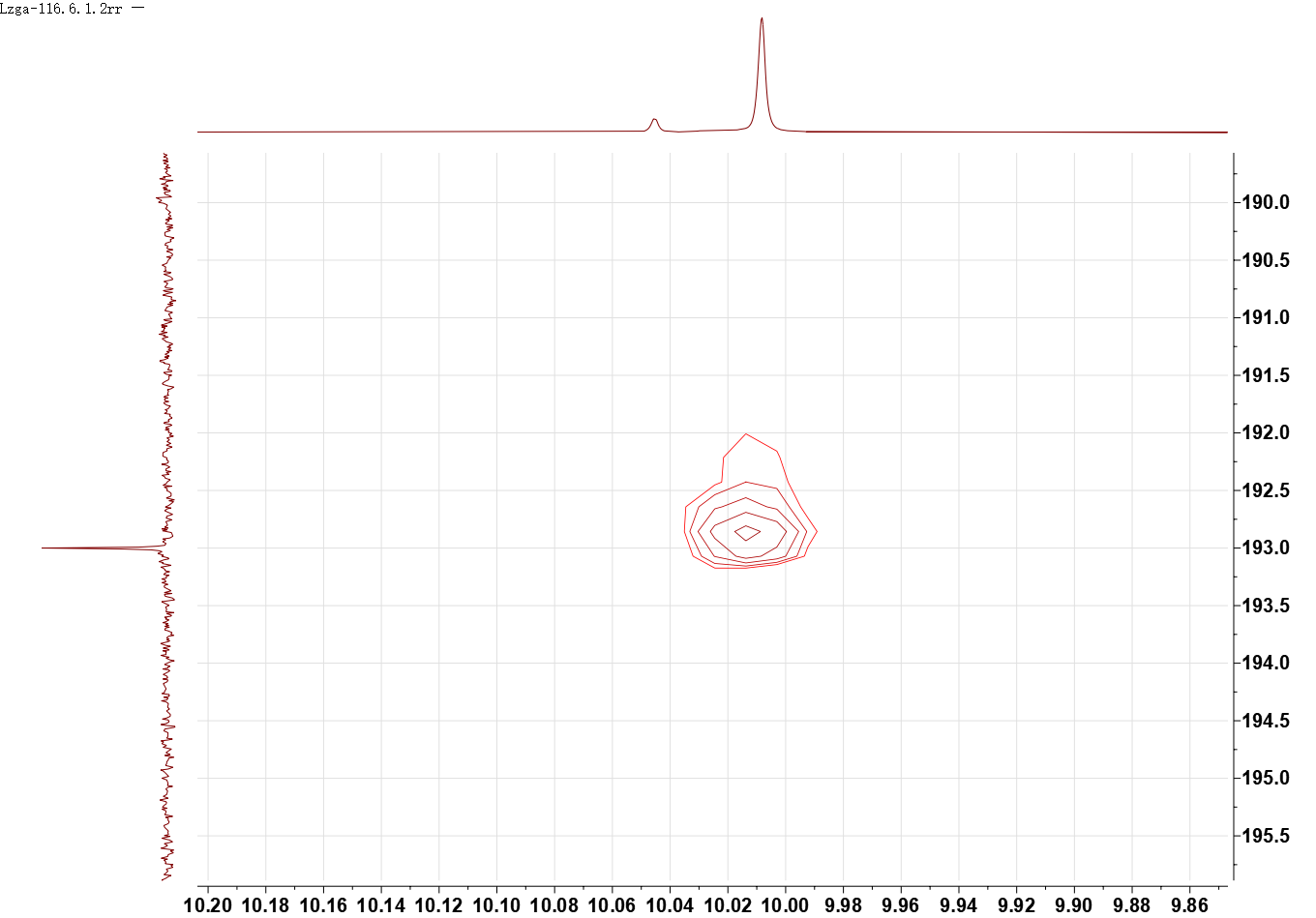


## Figure S35. ^1^H-^1^H COSY spectrum of **5**.


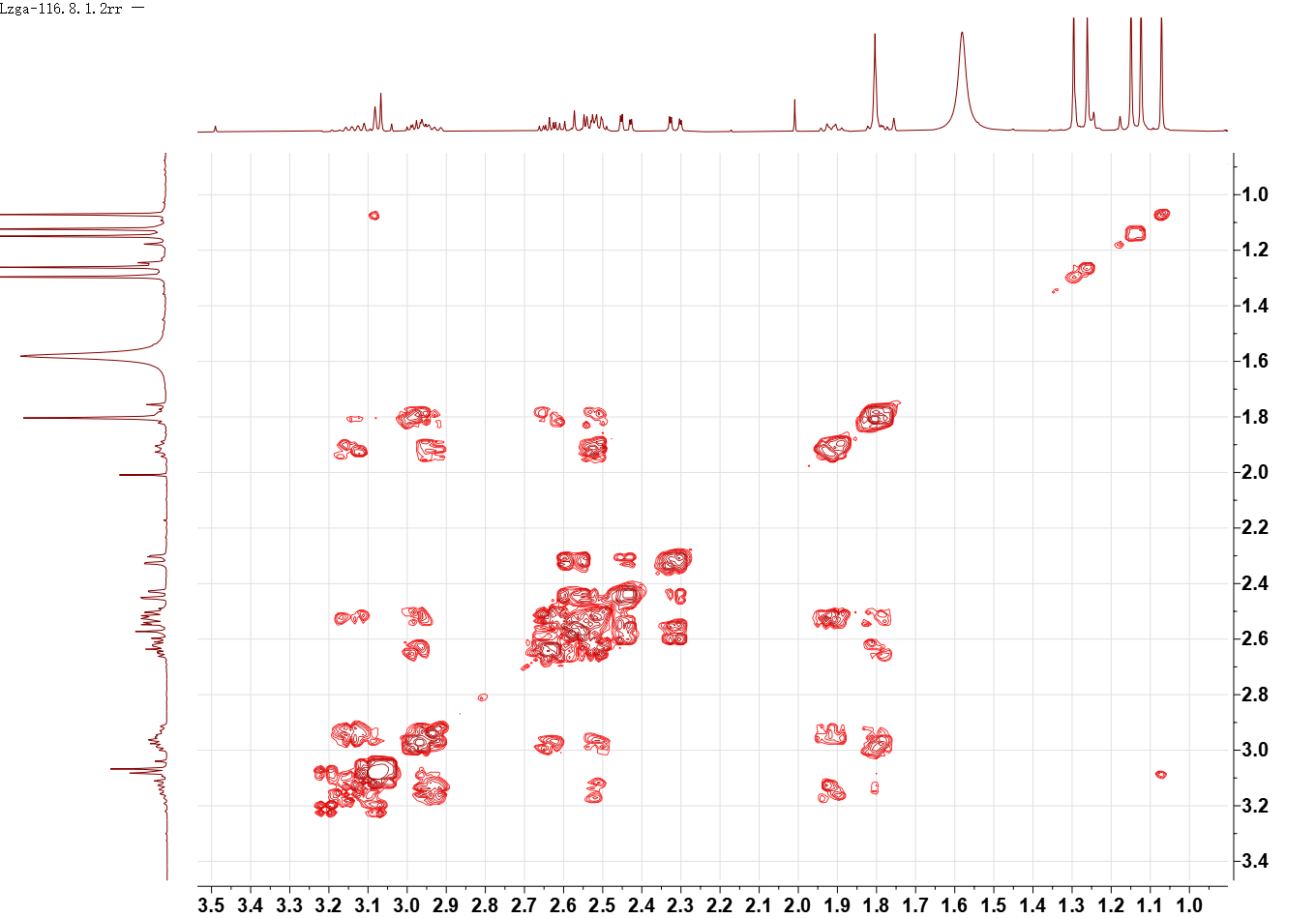


## Figure S36. HMBC spectrum of **5**.


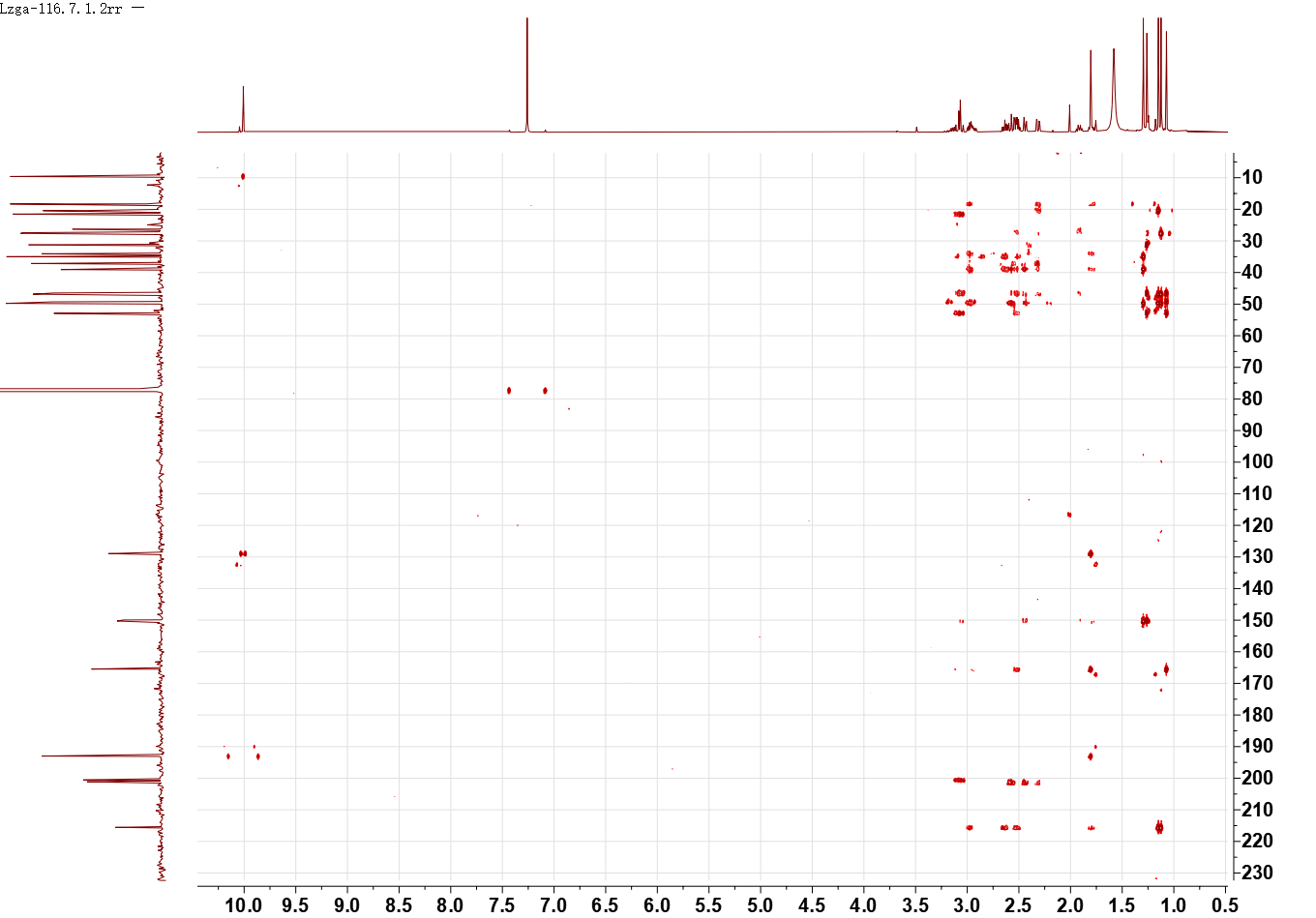


## Figure S37. ROESY spectrum of **5**.

## Figure S38. HRESIMS report of **5**.


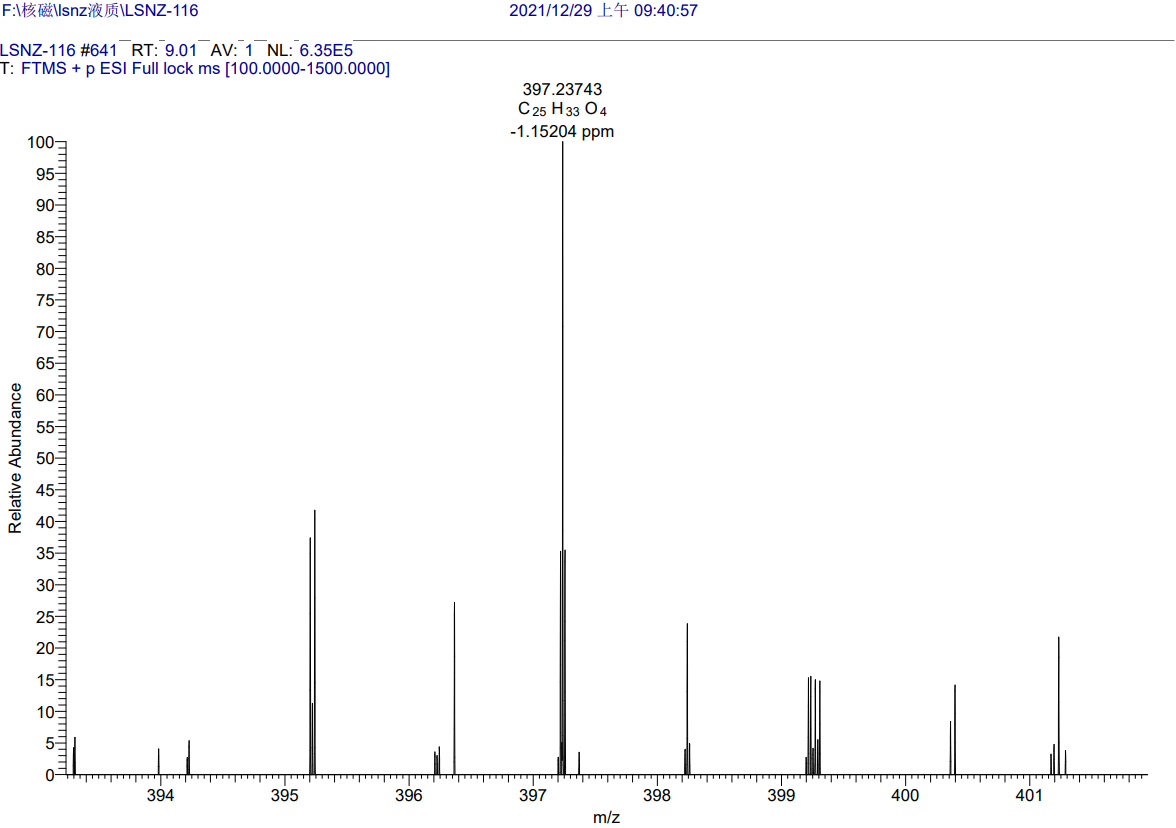


## Figure S39. ^1^H NMR spectrum of **6** (600 MHz, CDCl_3_).


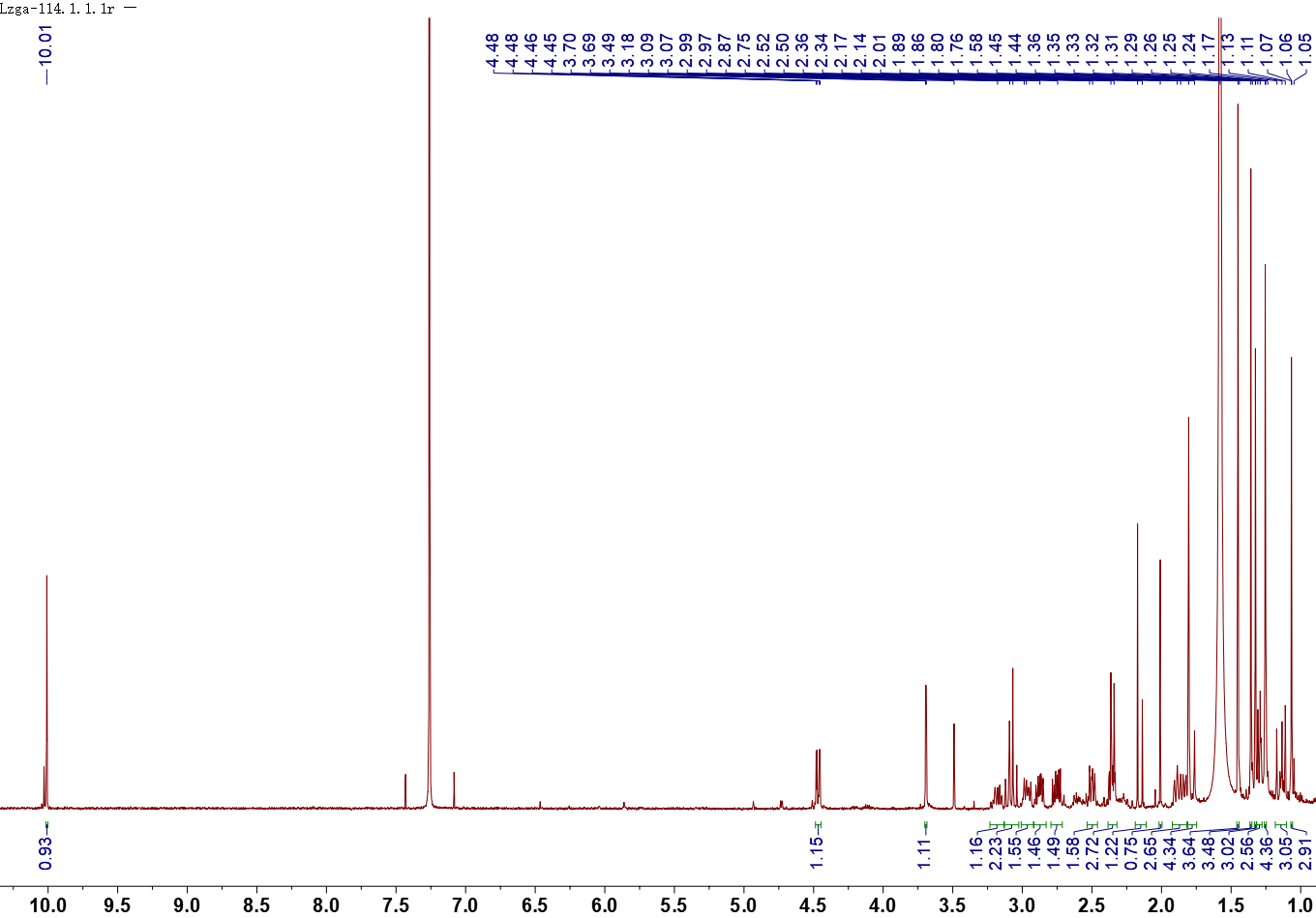


## Figure S40. ^13^C NMR and DEPT spectra of **6** (150 MHz, CDCl_3_).

## Figure S41. Enlarged HSQC spectrum (1.0-4.5) of **6**.


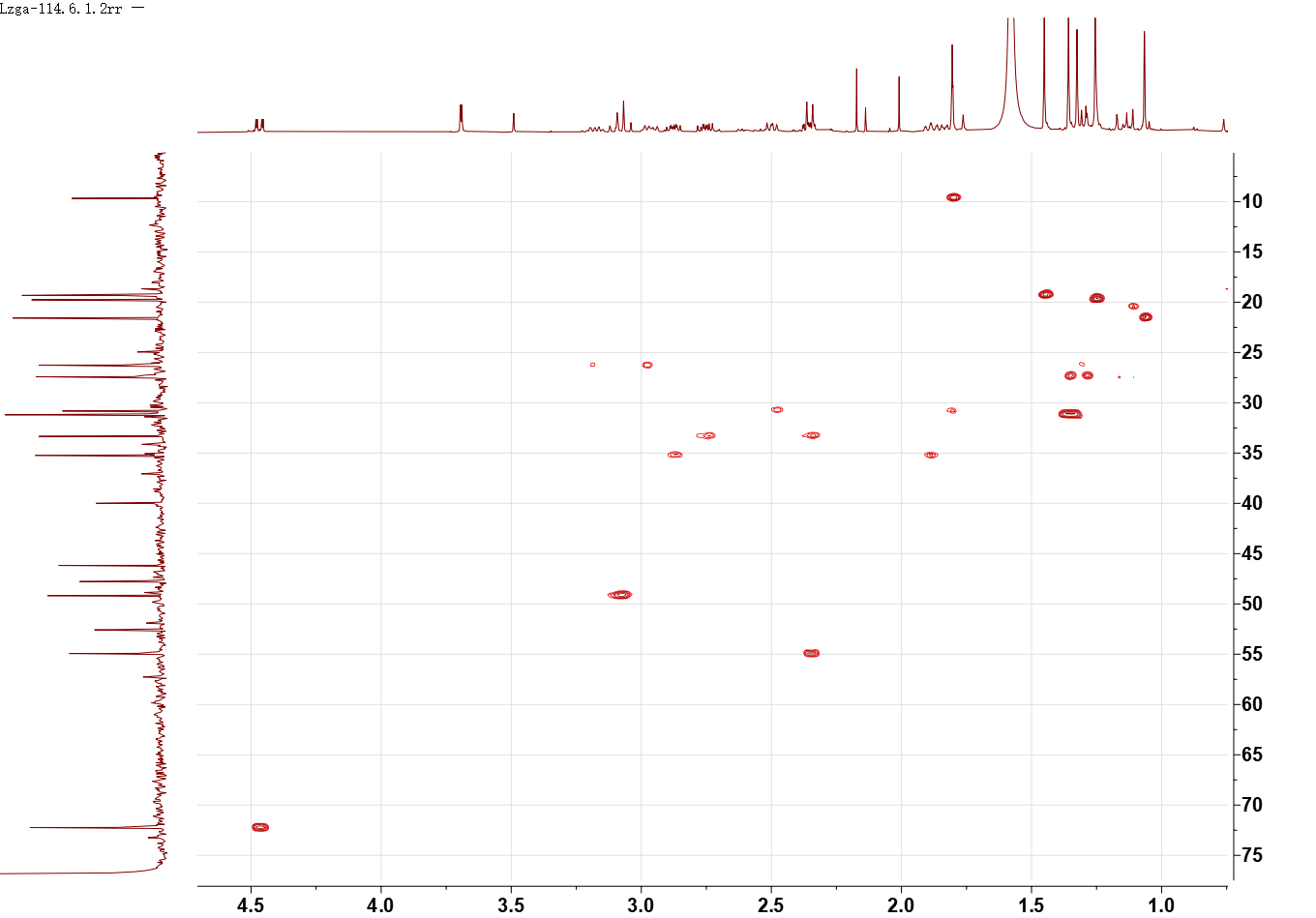


## Figure S42. Enlarged HSQC spectrum (9.8-10.1) of **6**.


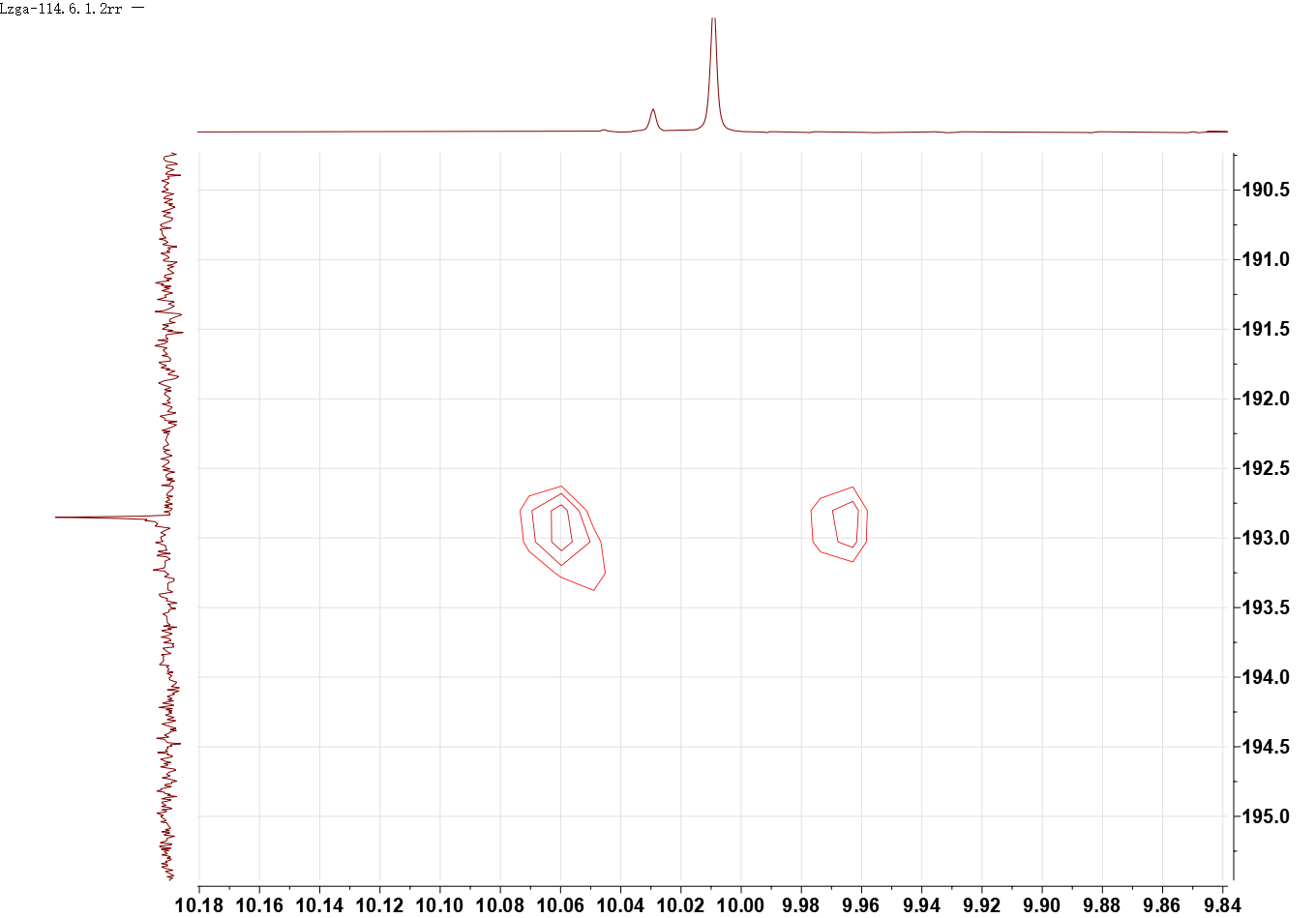


## Figure S43. ^1^H-^1^H COSY spectrum of **6**.


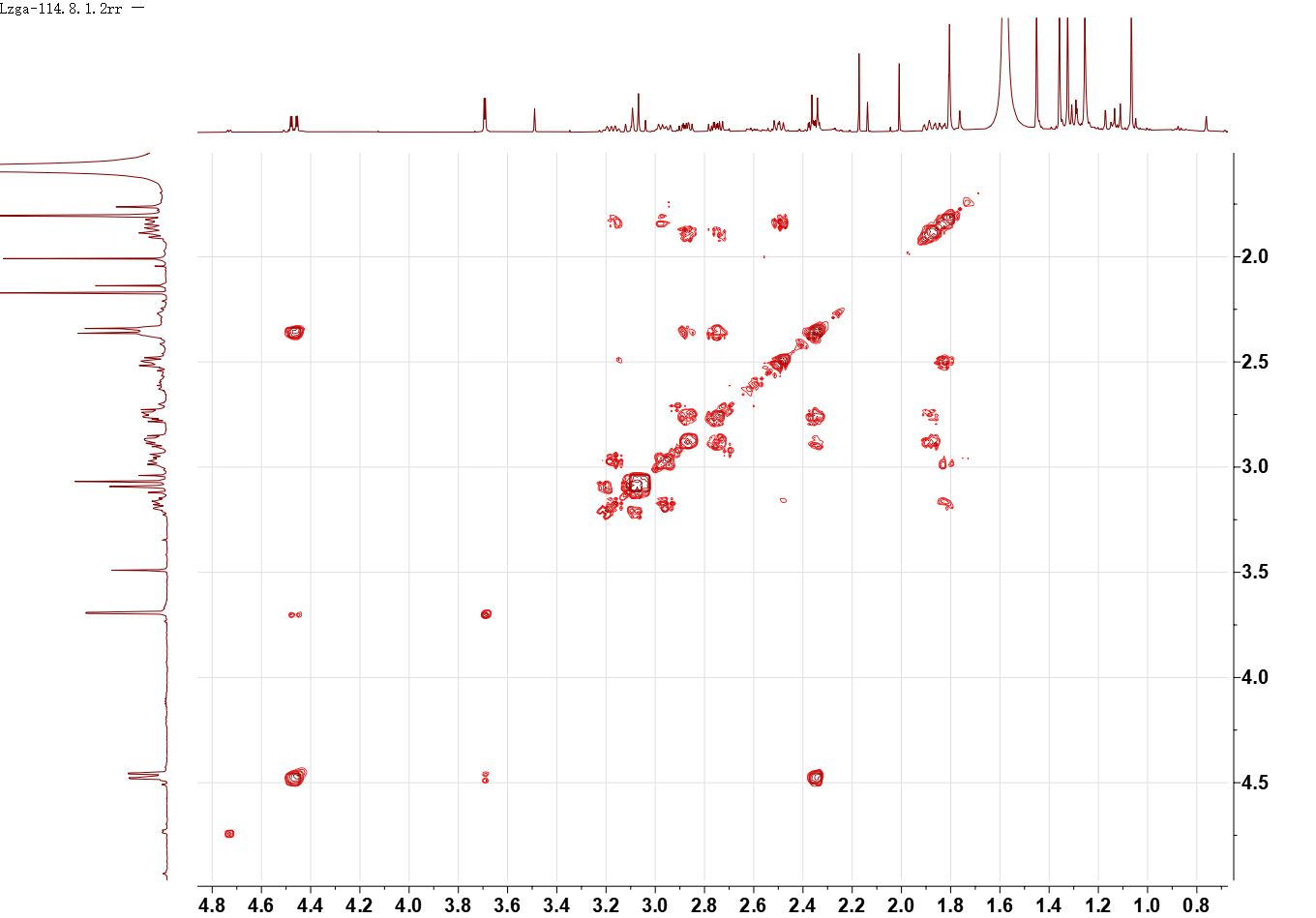


## Figure S44. HMBC spectrum of **6**.


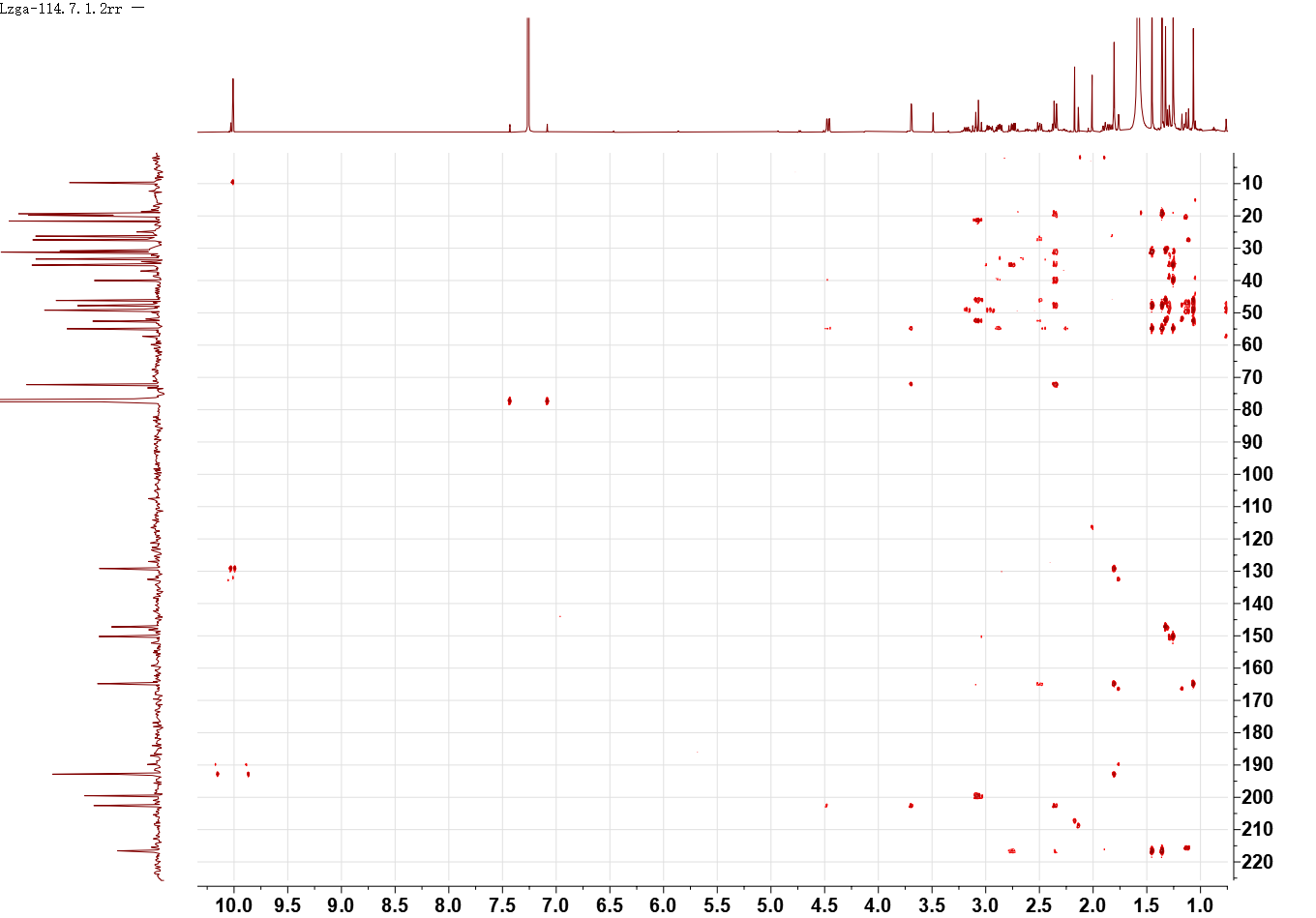


## Figure S45. ROESY spectrum of **6**.

## Figure S46. HRESIMS report of **6**.


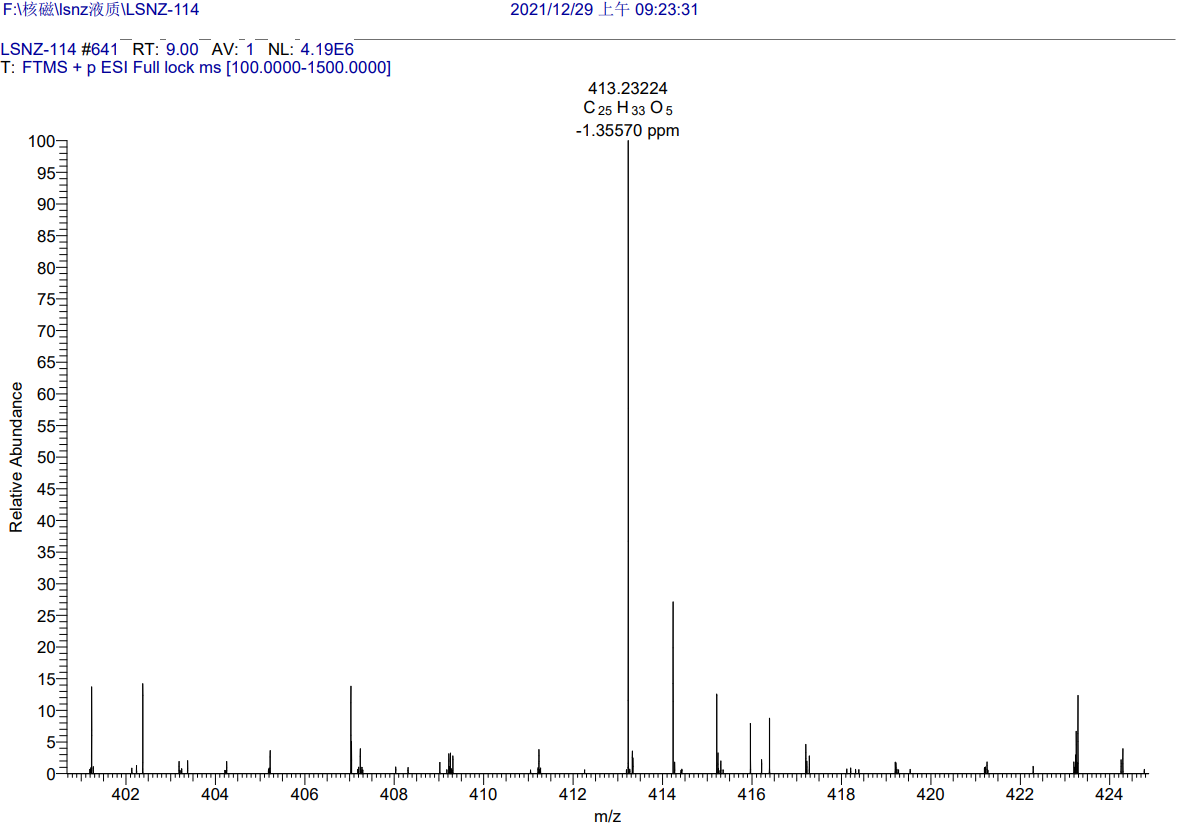


## Figure S47. ^1^H NMR spectrum of **7** (600 MHz, CDCl_3_)

## Figure S48. ^13^C and DEPT NMR spectra of **7** (150 MHz, CDCl_3_)

## Figure S49. HSQC spectrum of **7**.

## Figure S50. HMBC spectrum of **7**.

## Figure S51. ^1^H-^1^H COSY spectrum of **7**.

## Figure S52. ROESY spectrum of **7**.


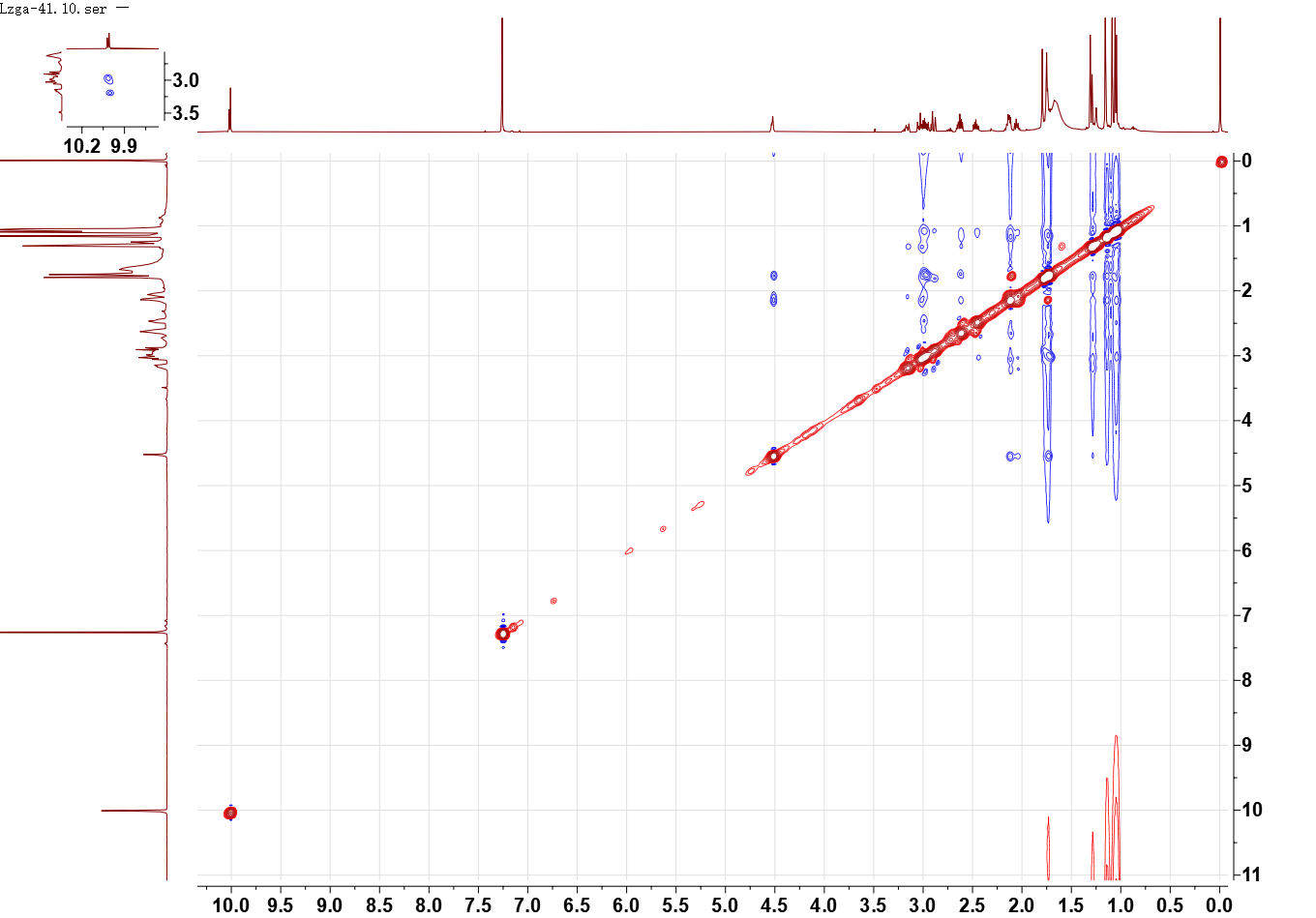


## Figure S53. HRESIMS of **7**.


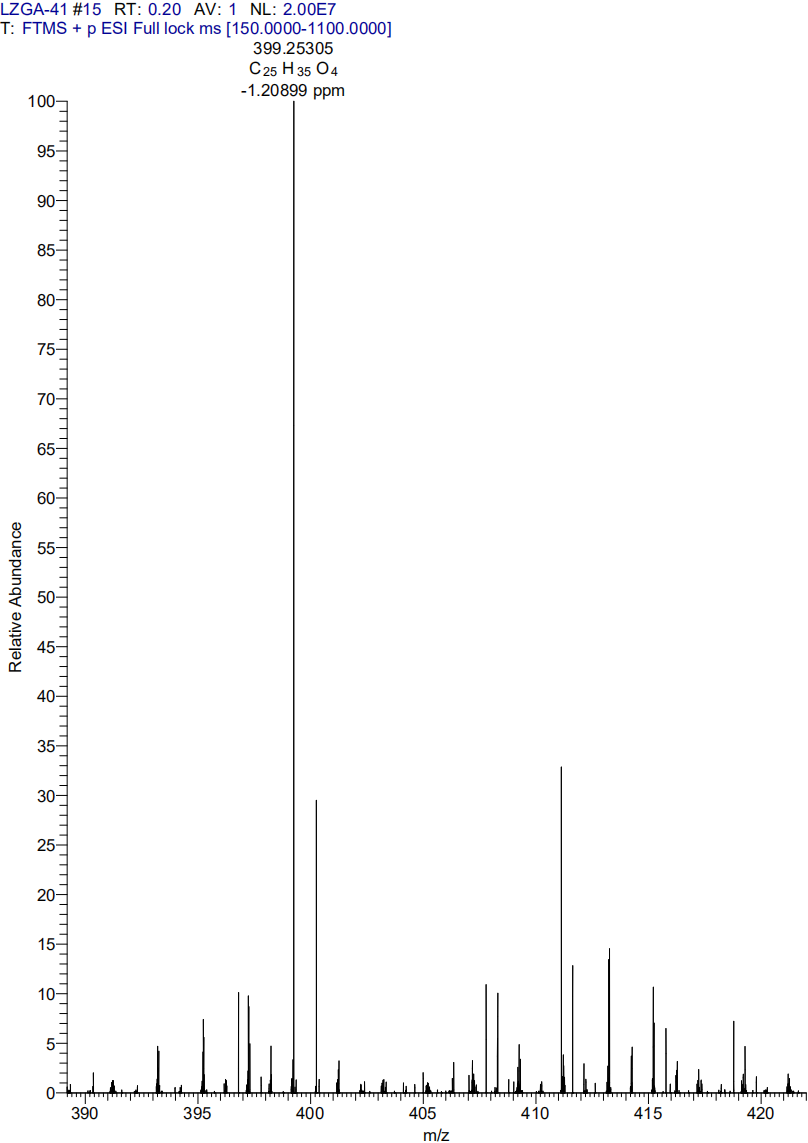


## Figure S54. ^1^H NMR spectrum of **8** (600 MHz, CDCl_3_)

## Figure S55. ^13^C and DEPT NMR spectra of **8** (150 MHz, CDCl_3_)

## Figure S56. HSQC spectrum of **8**.

## Figure S57. HMBC spectrum of **8**.

## Figure S58. ^1^H-^1^H COSY spectrum of **8**.

## Figure S59. ROESY spectrum of **8**.


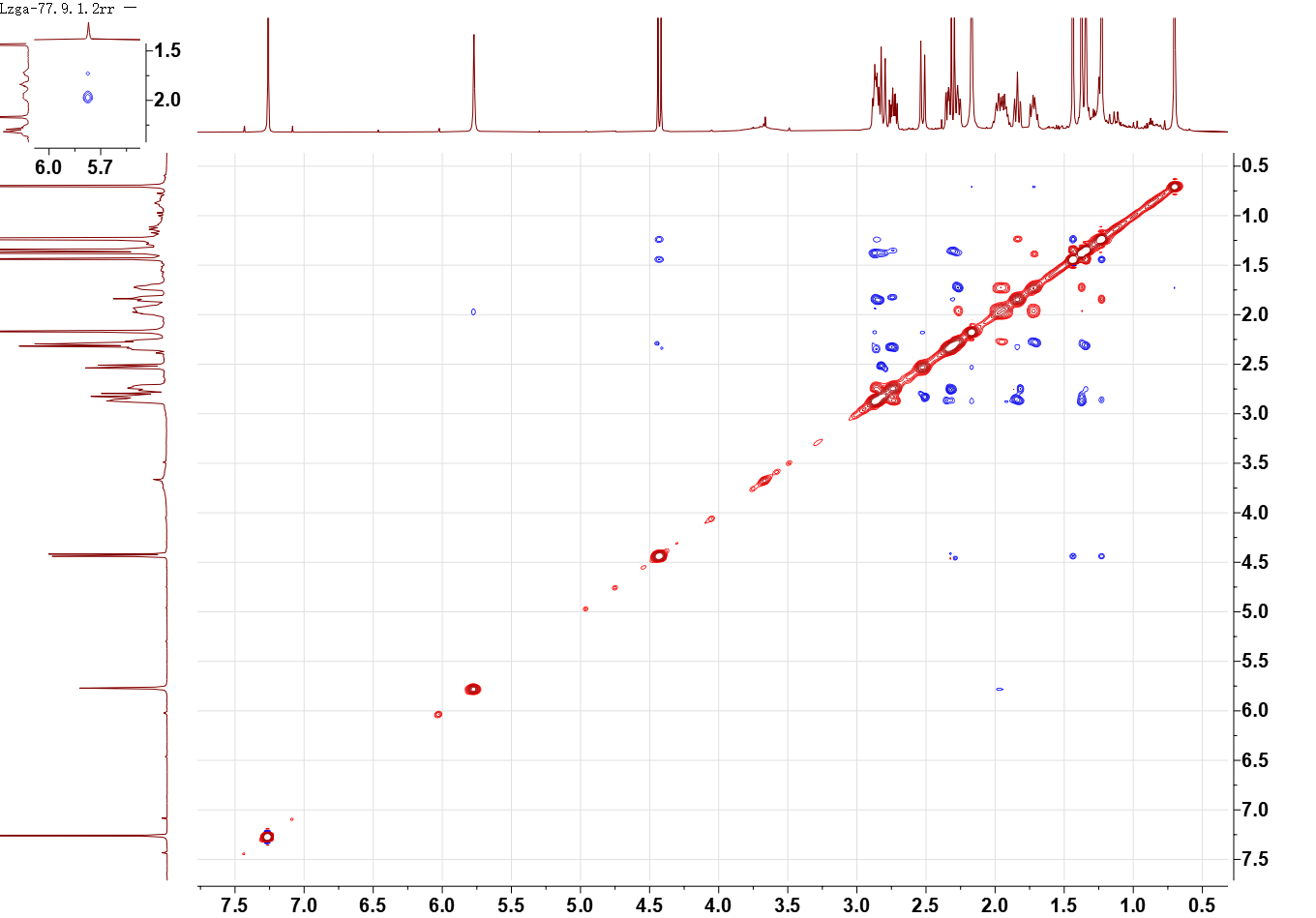


## Figure S60. HRESIMS of **8**.


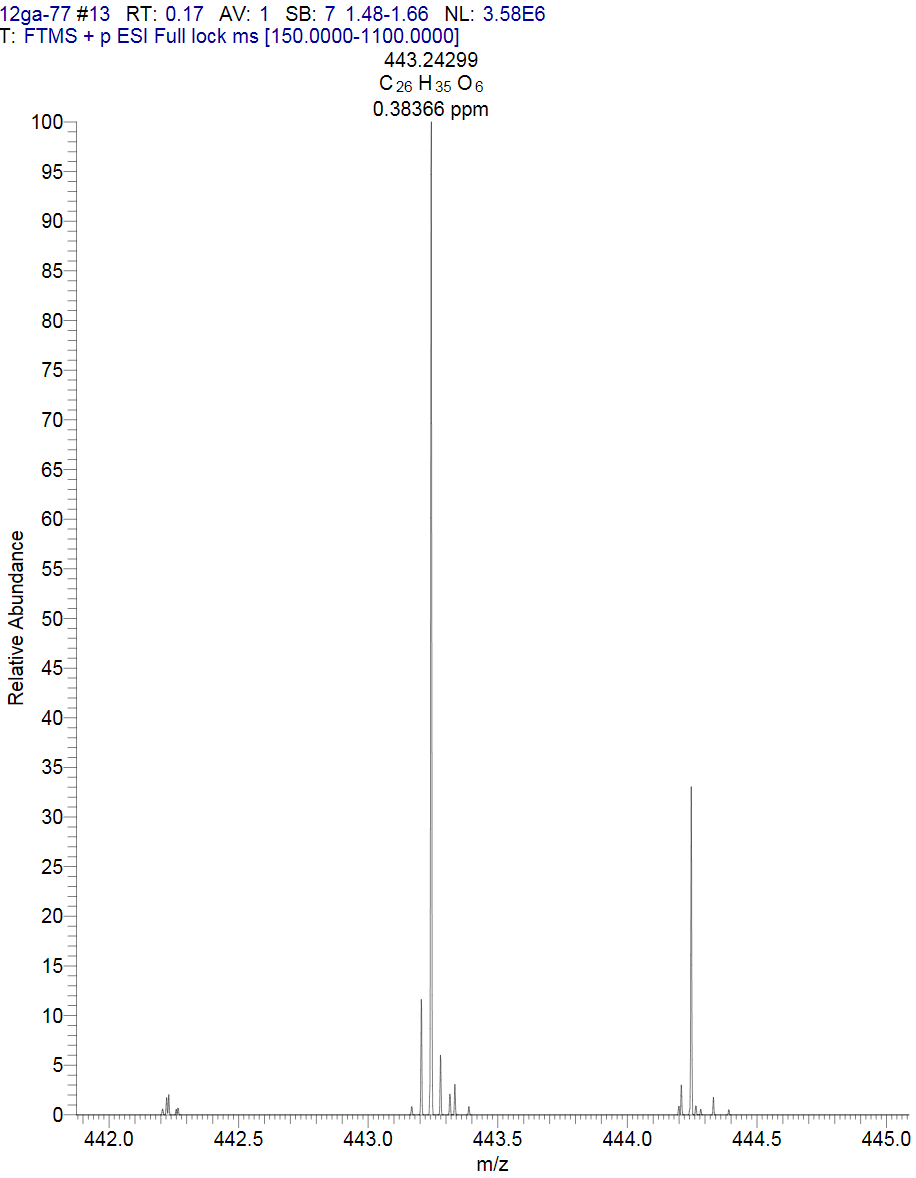


## Figure S61. ^1^H NMR spectrum of **9** (600 MHz, CDCl_3_)

## Figure S62. ^13^C and DEPT NMR spectra of **9** (150 MHz, CDCl_3_)

## Figure S63. HSQC spectrum of **9**.

## Figure S64. HMBC spectrum of **9**.

## Figure S65. ^1^H-^1^H COSY spectrum of **9**.

## Figure S66. ROESY spectrum of **9**.


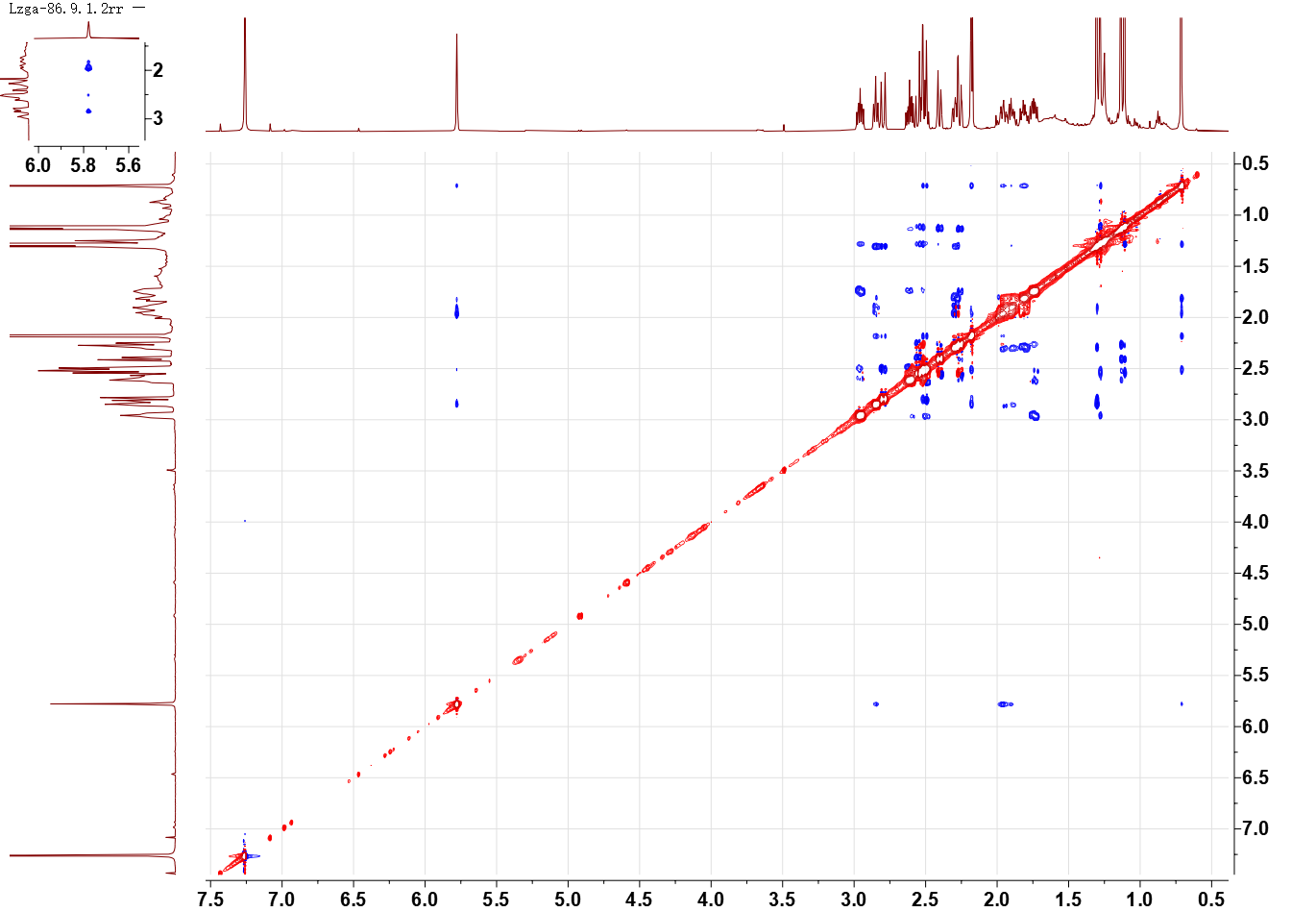


## Figure S67. HRESIMS of **9**.


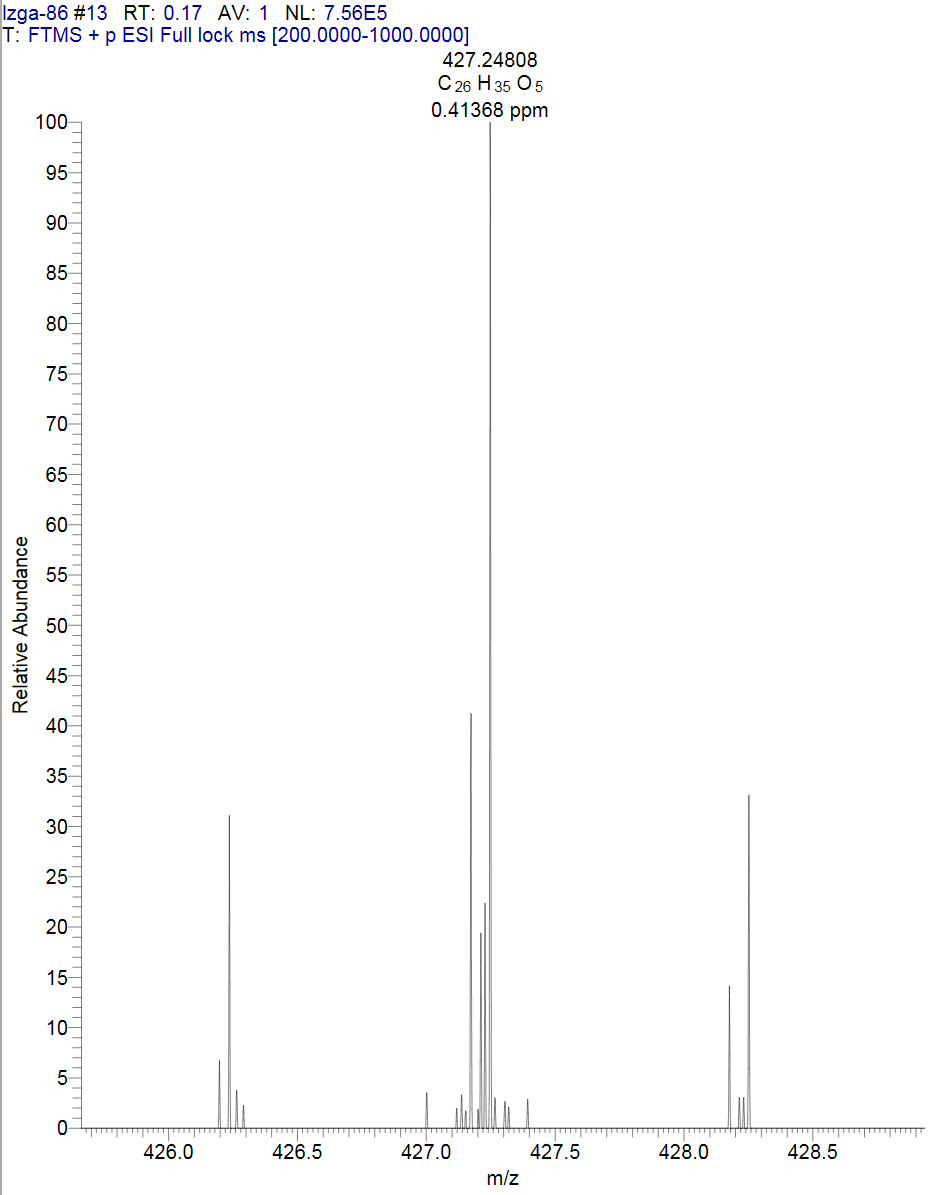


## Figure S68. ^1^H NMR spectrum of **10** (600 MHz, CDCl_3_)

## Figure S69. ^13^C NMR spectrum of **10** (200 MHz, CDCl_3_)

## Figure S70. HSQC spectrum of **10**.

## Figure S71. HMBC spectrum of **10**.

## Figure S72. ^1^H-^1^H COSY spectrum of **10**.

## Figure S73. ROESY spectrum of **10**.


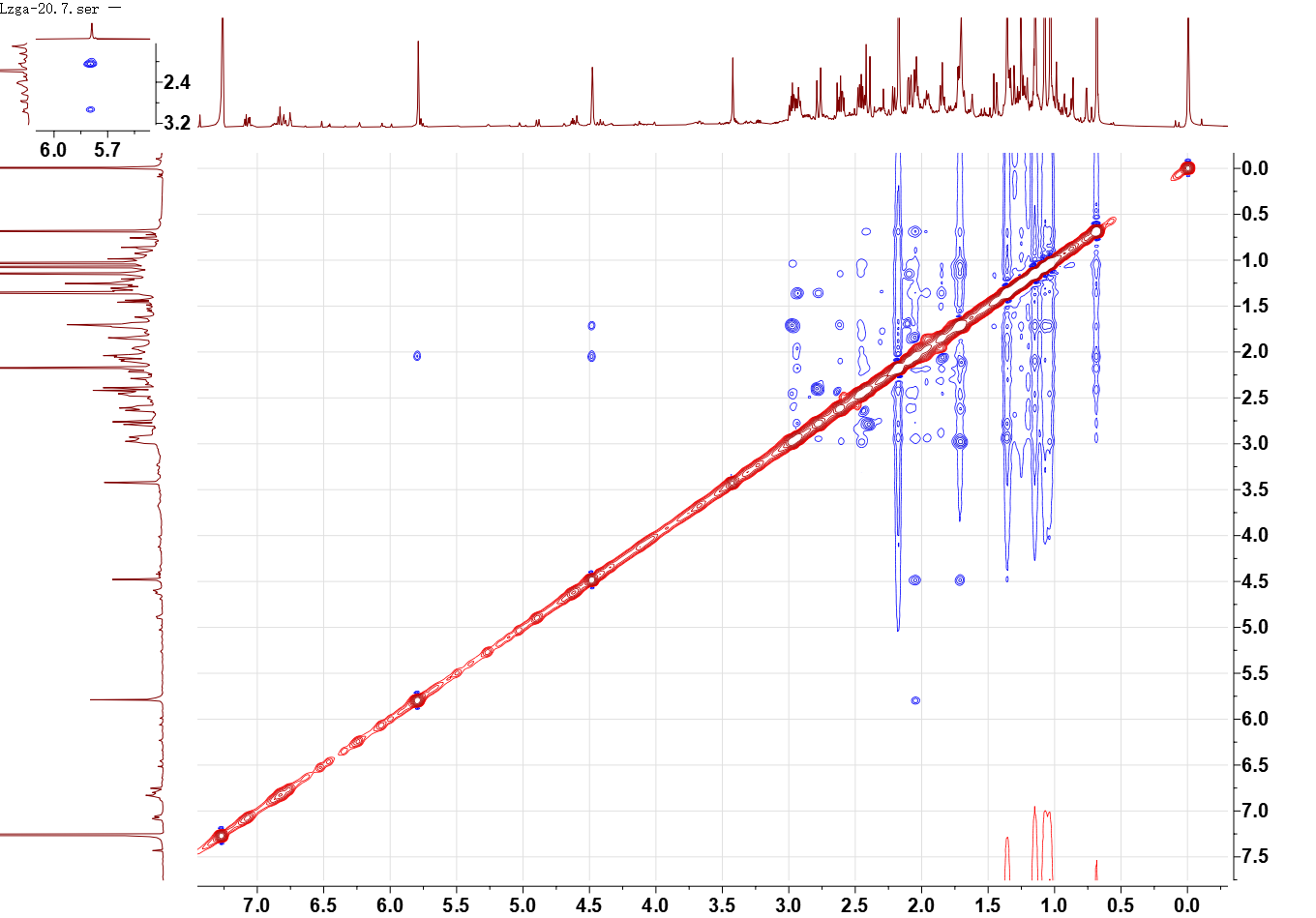


## Figure S74. HRESIMS of **10**.


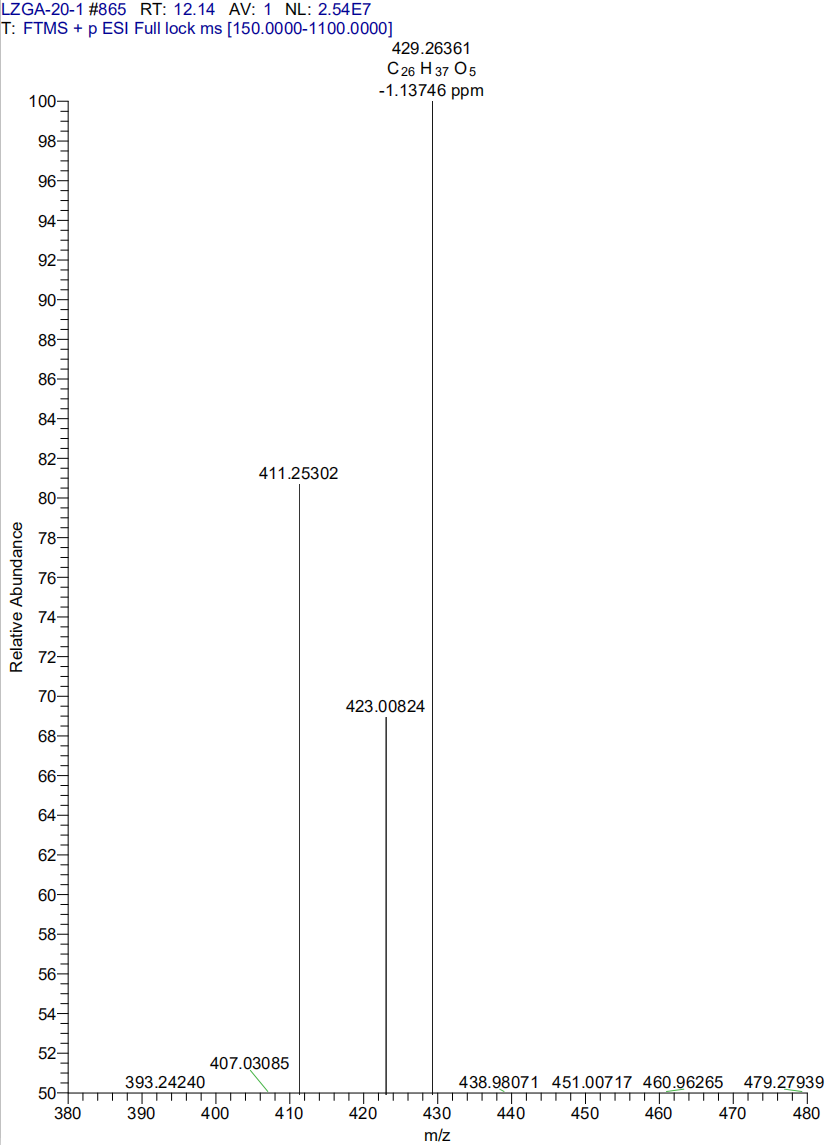

Supplement: Supplementary file 1 — Additional file 1. The NMR, HRESIMS spectra of compounds 1–10 [file 13659_2022_356_MOESM1_ESM.docx]
